# Palladium complexes containing imino phenoxide ligands: synthesis, luminescence, and their use as catalysts for the ring-opening polymerization of *rac*-lactide

**DOI:** 10.1007/s00706-017-2119-1

**Published:** 2017-12-12

**Authors:** Mrinmay Mandal, Manuela List, Ian Teasdale, Günther Redhammer, Debashis Chakraborty, Uwe Monkowius

**Affiliations:** 1 Institute of Inorganic Chemistry, Johannes Kepler University Linz, Altenbergerstr. 69, 4040 Linz, Austria; 2 School of Chemical and Biomolecular Engineering, Georgia Institute of Technology, Atlanta, GA 30332-0100 USA; 3 Institute for Chemical Technology of Organic Materials, Johannes Kepler University Linz, Altenbergerstr. 69, 4040 Linz, Austria; 4 Institute of Polymer Chemistry, Johannes Kepler University Linz, Altenbergerstr. 69, 4040 Linz, Austria; 5 Materialwissenschaften und Physik, Abteilung für Mineralogie, Paris-Lodron Universität Salzburg, Hellabrunner Str. 34, 5020 Salzburg, Austria; 6 Department of Chemistry, Indian Institute of Technology Madras, Chennai, Tamil Nadu 600 036 India; 7 Linz School of Education, Johannes Kepler University Linz, Altenbergerstr. 69, 4040 Linz, Austria

**Keywords:** Imino phenoxide, Palladium, Crystal structure, ROP, *rac*-Lactide, Luminescence

## Abstract

**Abstract:**

The preparation, structural characterization, luminescence, and catalytic activity of three palladium(II) complexes bearing imino phenoxide ligands are reported. The X-ray studies revealed that the complexes are mononuclear with palladium centres coordinated in a square-planar coordination environment. Two of the complexes are emissive in solution at room temperature. The catalytic activities towards the ring-opening polymerization of *rac*-lactide (*rac*-LA) were tested. Polymers with moderate molecular weights and relatively broad dispersity (*Ð*) were obtained. Kinetic studies revealed that the polymerization followed first-order kinetics.

**Graphical abstract:**

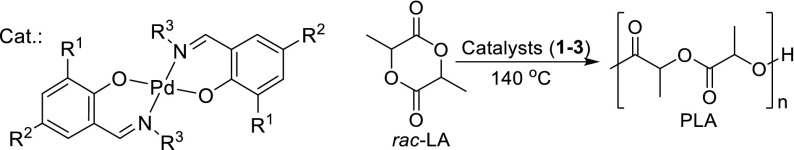

**Electronic supplementary material:**

The online version of this article (10.1007/s00706-017-2119-1) contains supplementary material, which is available to authorized users.

## Introduction

Tetradentate salen-type ligands are omnipresent in coordination chemistry. They are easy to synthesize and they exhibit a high structural variability due to a large number of commercial available building blocks which can be used as starting materials. In many cases, they form highly stable metal complexes throughout the whole periodic table and a plethora of metal complexes have been reported in the past [1, 2]. Related imino phenoxide ligands could be considered as half salen-type ligands and are somewhat less frequently used as ligands. They also exhibit similar advantageous coordination modes, i.e. the combination of a moderate hard and a hard donor atom with an anionic nature of the ligand. Hence, reports from almost all areas of coordination chemistry have been published up to now. Such complexes have found applications in catalysis or biochemistry or feature interesting structural, magnetic, photophysical or electrochemical properties [3–12].

Besides ubiquitous applications in catalysis for coupling reactions, palladium complexes were intensively investigated due to their interesting luminescence properties [13, 14]. However, only limited number of papers has been published investigating emissive properties using salen-type or imino phenoxide ligands [15]. Palladium in its oxidation state +2 has a d^8^ configuration and hence prefers a square-planar coordination. It is well known that such complexes form polymeric columnar structures, often with infinite and close Pd–Pd bonds. Contrary to their platinum congeners [16], such complexes usually do not feature luminescence from excited states based on these metallophilic interactions. However, Pd(II) complexes bearing ligands with energetic low-lying π*-accepting orbitals exhibit phosphorescence from either metal-to-ligand charge transfer (^3^MLCT) or intraligand (^3^IL) excited states [17].

In recent publications we have shown that main group as well as transition metal imino phenoxide complexes can be used as polymerization catalysts, e.g. for the preparation of polyesters like poly(lactic acid) (PLA) and poly(caprolactone) (PCL) [18–23]. They constitute an interesting family of environmentally benign biodegradable polymers with a wide variety of potential applications in the biomedical area or as alternatives to persistent polyolefin materials [24–29]. Usually, aliphatic polyesters are prepared by the ring-opening polymerization (ROP) of cyclic esters using metal-based initiators, particularly of toxic tin containing compounds. In the past years, much effort has been spent in studies with alternative catalyst systems containing other, partly less toxic metals [30–37].

In this contribution, we report on the synthesis, structural characterization, photophysical, and catalysis studies of some square-planar Pd(II) complexes bearing imino phenoxide ligands with different structural modifications. Furthermore, although similar Pd(II) complexes have been prepared previously, to the best of our knowledge, the catalysis for the ROP of *rac*-LA using Pd(II) complexes containing the imino phenoxide backbone is still unreported [38–43].

## Results and discussion

### Synthesis and characterization

The ligands were prepared following a reported literature procedure [44, 45]. The complexes were synthesized by mixing an ethanolic solution of the Schiff base and Pd(II) acetate in refluxing ethanol (Scheme 1).

**Figure:**
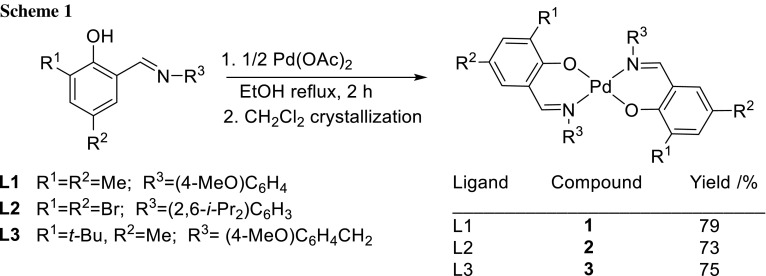

Complexes **1–3** were isolated in high yields. Their identity and purity are supported by elemental analysis, NMR spectroscopy, and mass spectrometry. In the ESI-MS spectra, signals representing the ions [L_2_PdH]^+^ and [L_2_PdNa]^+^ are detected. The IR spectra of the free ligands show intense signals in the region from 1623 to 1632 cm^−1^ (Fig. S10). In the complexes, the spectra are shifted towards lower frequencies (1607–1624 cm^−1^), which eventually revealed the coordination of imine nitrogen atoms to the metal (Fig. S11). In the ^1^H NMR spectra, the formation of the metal complexes is shown by the disappearance of the phenol –OH signals of the ligands. In addition, the signals corresponding to the protons of the CH=N groups appear low field shifted (7.52–7.59 ppm) compared to those of the ligands (8.4–8.6 ppm) which indicates the coordination of nitrogen and oxygen atoms of the ligands to the metal.

### Structural studies

**Fig. 1:**
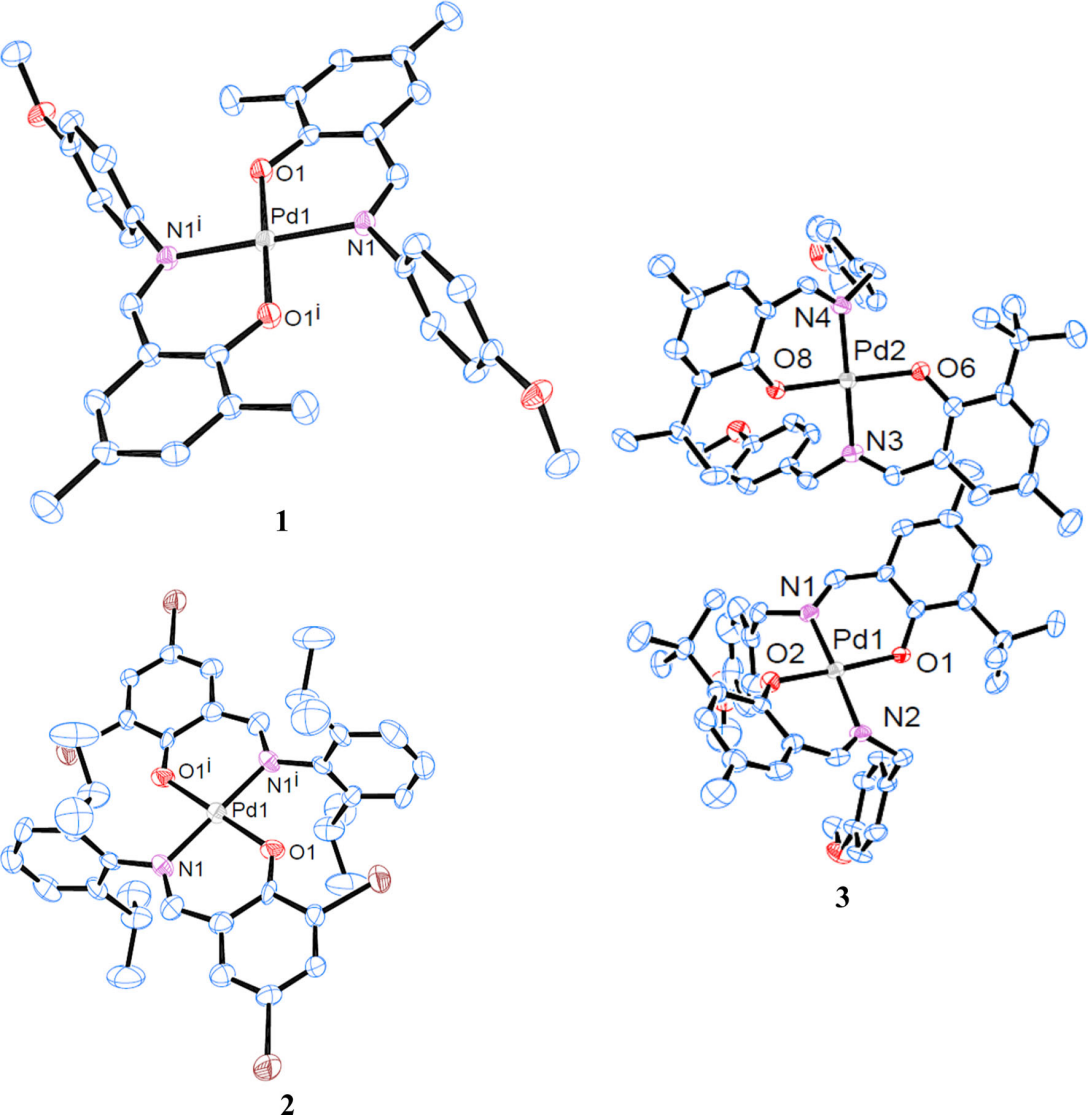
Molecular structures of **1–3**; thermal ellipsoids were drawn at 50% probability level. Hydrogen atoms were removed for clarity

Single crystals suitable for X-ray diffraction of all three complexes were grown by slow evaporation of the solvent from their solutions in dichloromethane over a period of 1 week. All complexes were obtained as red crystalline solids. The crystallographic data are summarized in Table 4. Molecular structures and selected bond lengths and bond angles are displayed in Fig. 1 and Table 1, respectively. Complex **1** crystallizes in the monoclinic space group *P*2_1_/*n*, complex **2** in the triclinic space group *P* $\bar{1}$, both with one half formula units in the asymmetric unit with the palladium atom as the inversion centre. Complex **2** is isostructural to the homologues copper(II) complex [12]. The palladium atom is coordinated by two nitrogen and two oxygen atoms with an angular sum of exact 180° indicative for a perfect planar coordination. The coordination geometry of **2** is further stabilized by C–Br/π-interactions: the bromine atom points directly to π-system of the phenyl-group of the aniline moiety with a distance between the ring plane and the bromine atom of ~ 3.44 Å which is typical for this kind of interactions [46, 47]. Similarly, for **1** the methyl-substituents are engaged in C–H/π-interactions. **3** is monoclinic, *P* $\bar{1}$ with two complexes per asymmetric unit. Contrary to **1** and **2**, the palladium atom in **3** exists in a strongly distorted square-planar coordination environment [angular sum 362.0° (Pd1)/361.1° (Pd2)]. This might be due to steric reasons and packing effects. The palladium atoms in all complexes are not engaged in any further Pd–Pd interactions.

**Table 1:** Selected bond lengths/Å and bond angles/° for **1**–**3**

	**1**	**2**	**3**
Pd–N	2.021(2)	2.020(12)	2.028(6) 2.035(7) 1.996(5) 2.021(5)
Pd–O	1.975(2)	1.986(11)	1.982(5) 1.982(5) 1.996(5) 2.005(5)
O–Pd–N^a^	92.22(9)	92.8(5)	90.6(2)/90.4(2) (Pd1) 90.4(2)/89.6(2) (Pd2)
O–Pd–N	87.78(9)	87.2(5)	90.1(2)/90.4(2) (Pd1) 90.9(2)/89.7(2) (Pd2)

^a^Bite angle

### Electronic spectra

**Fig. 2:**
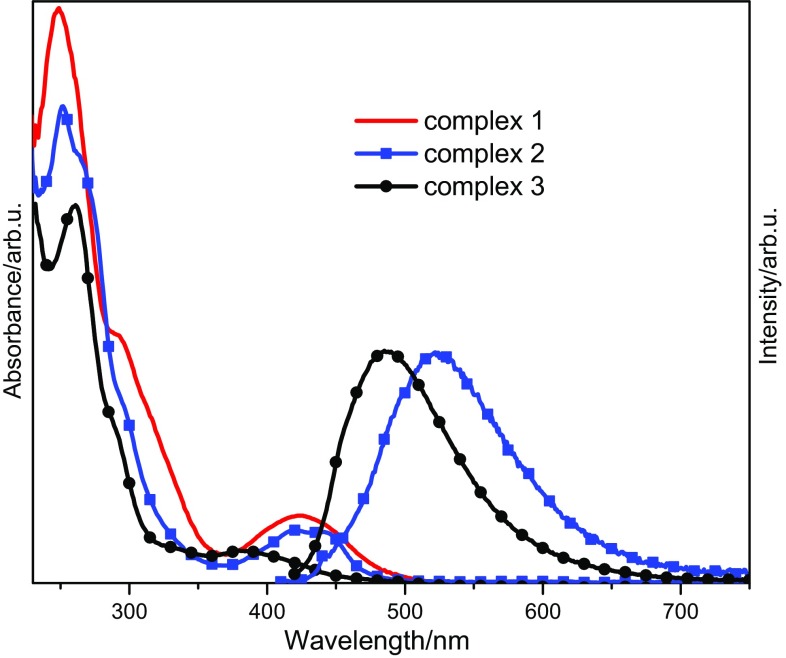
UV–Vis absorption and emission spectra of **1**–**3** in DCM at room temperature

The electronic spectra of all the complexes were recorded in solution at room temperature (Fig. 2). The data are summarized in Table 2. The absorption spectra are very similar to the reported ones [39, 40, 42]. The presence of bands below 300 nm is assigned to π–π* transition of the ligands. The long wavelength absorptions can be assigned to an MLCT. Related Pd(II) complexes bearing salen-type ligands are sometimes only weakly emissive. An recent theoretical study on Pd(II) salen complexes explains the poor emissive behaviour by a low energy gap between emissive MLCT and quenching dd excited states [15]. The authors suggest a substitution of the ligands with strongly electron-withdrawing ligands. Indeed, the ligand in complex **2** carries the moderately electron-withdrawing bromine substituent, whereas the ligand in the non-emissive complex **1** is substituted by a strongly electron-donating methoxy group. For **3**, the ligand is substituted with a very bulky *t*-butyl group which leads not only to a strongly distorted coordination geometry as elucidated by the solid state structure, but also to a more rigid structure with respect to a distortion in the excited state. Minimizing the excited state structural distortion has been found to be effective in increasing the luminescence quantum yield. It should be noted that analogous Pt-complexes often feature intensive luminescence [14].

**Table 2:** UV/Vis spectroscopic data of the complexes **1**–**3**

Compound	Absorption *λ*/nm [log(*ε*/dm^3^ mol^−1^ cm^−1^)]	Emission/nm
**1**	247 (4.77), 293(4.41), 423 (3.84)	–
**2**	252 (4.79), 267 (sh, 4.73), 295 (sh, 4.38), 420 (3.83), 431 (3.82)	522
**3**	261 (4.75), 289 (sh, 4.37), 334 (3.72), 385 (3.66)	487

### Polymerization studies

**Fig. 3:**
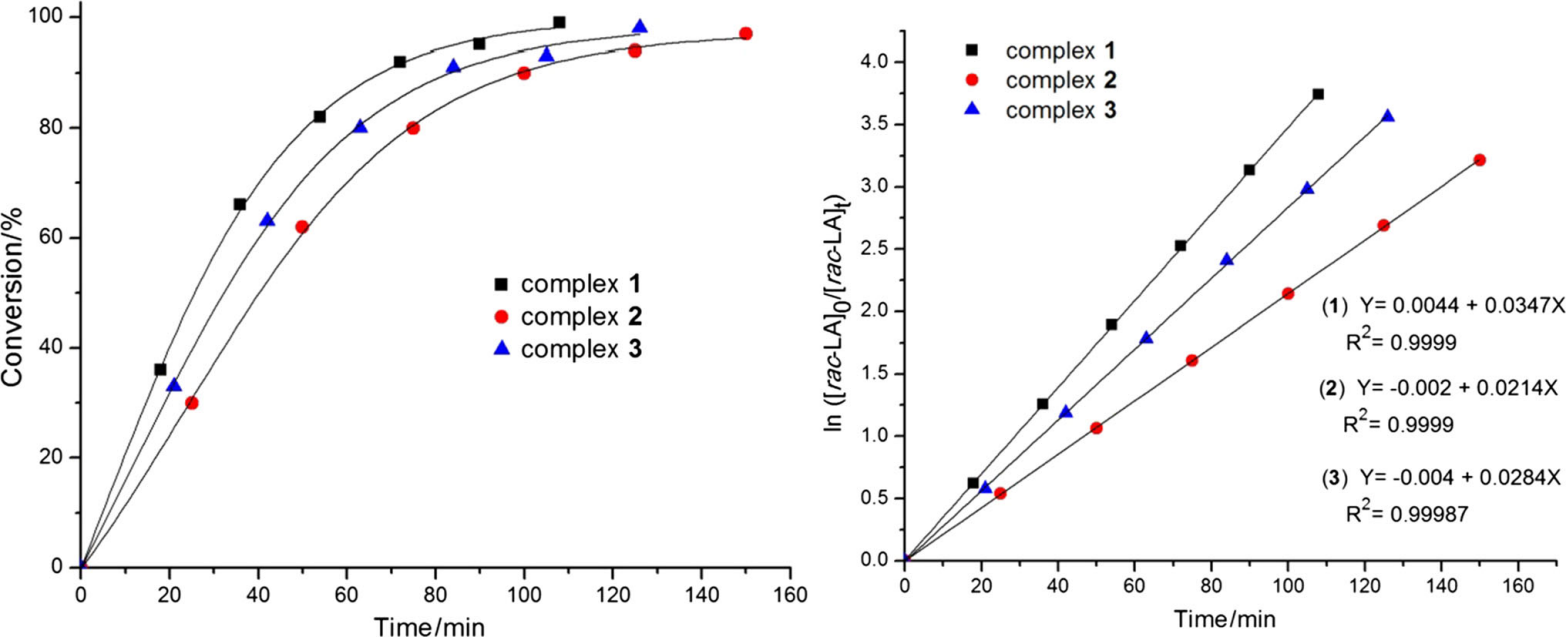
*rac*-LA conversion vs. time (left) and ln ([*rac*-LA]_0_/[*rac*-LA]_t_) vs. time plot (right) using **1**–**3**: [*rac*-LA]_0_:[Cat]_0_ = 200:1 at 140 °C

The complexes **1–3** were investigated as catalysts for ROP of *rac*-LA (Scheme 2). The kinetics of polymerization was examined by ^1^H NMR spectroscopy. The polymerization was performed in a 200:1 ratio ([*rac*-LA]_0_/[Cat]_0_ = 200:1) catalysed by complexes **1–3**. The plot of % conversion of *rac*-LA against time gives a sigmoid curve meaning that initially the conversion rate is high but decreases significantly at later stage (Fig. 3, left). There is a linear correlation of the values in the semi-logarithmic plot of ln([*rac*-LA]_0_/[*rac*-LA]_t_) vs. time indicative for a first-order dependency on the monomer concentrations with the absence of any induction period (Fig. 3, right). This result suggests that the active species remained unchanged and active during the entire course of polymerization. The apparent rate constants (*k*_app_) were extracted from the linear plot of ln([*rac*-LA]_0_/[*rac*-LA]_t_) vs. time and were found to be 3.47 × 10^−2^ min^−1^, 2.14 × 10^−2^ min^−1^, and 2.84 × 10^−2^ min^−1^ for **1**–**3**, respectively (Fig. 2, right), i.e. the activities of the catalysts are slightly higher than those found for the copper(II) homologue complexes we reported previously [12].

**Figure:**
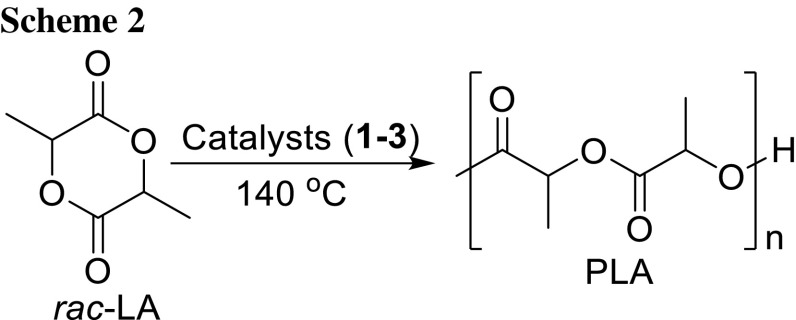

Furthermore, bulk polymerizations were performed in a 200:1 (monomer:catalyst) ratio at a temperature of 140 °C and the polymerization was terminated once the rise in the viscosity was observed and the stirring finally ceased. In these experiments, all complexes exhibits a comparable activity to that already found for the kinetic studies above. The representative results are displayed in Table 3. All complexes yielded polymers with moderate molecular weight (*M*_n_) and relatively broad dispersities (*M*_w_/*M*_n_ = 1.91–1.98). The large difference between the experimental and theoretic values and broad *Ð* compared to literature values [48] are thought to be due to the occurrence of polymerization side reactions such as inter-molecular or intra-molecular transesterification as well as a slow initiation rate compared with a fast propagation [49–52].

**Table 3:** Polymerization data for *rac*-LA using **1**–**3** in 200:1 (monomer:catalyst) ratio at 140 °C

Entry	Catalyst	Yield/%	Time^a^/min	*M*_n_^obs^/kg mol^−1^	*M*_w_/*M*_n_	*k*_app_/10^−2^ min^−1^
1	**1**	99	109	12.89	1.91	3.47
2	**2**	97	153	10.02	1.98	2.14
3	**3**	98	128	10.54	1.94	2.84

^a^Time of polymerization measured by quenching the polymerization reaction when stirring ceased

## Conclusion

In conclusion, we have synthesized three new palladium complexes containing imino phenoxide ligands. The single-crystal X-ray analysis revealed that the palladium atom is surrounded by two ligands in a square-planar coordination environment. Complex **2** and **3** are emissive in solution at room temperature. These compounds have catalytic activity towards the polymerization of *rac*-LA. The *M*_n_ of the polymers is moderate with relatively broad *M*_w_/*M*_n_ values.

## Experimental

All manipulations were carried out in an atmosphere of dry nitrogen using standard Schlenk techniques. CDCl_3_ used for NMR spectral measurements was dried over calcium hydride for 48 h, distilled and stored in a glove box. ^1^H and ^13^C{^1^H} NMR spectra were recorded in Bruker Digital Avance III (300 MHz) instrument. Chemical shifts for ^1^H was referenced to residual solvent resonances and are reported as parts per million relative to SiMe_4_. IR spectroscopy was performed on a Shimadzu IRAffinity-1 FTIR spectrophotometer that was equipped with a Specac Golden GateTM single-reflection diamond ATR accessory. Mass spectra were collected on a Finnigan LCQ DecaXPPlus ion trap mass spectrometer with an ESI ion source. Elemental analyses were carried out at the Institute for Chemical Technology of Organic Materials at Johannes Kepler University Linz. MALDI-TOF measurements were performed on a Bruker Daltonics instrument in a dihydroxybenzoic acid matrix. For photophysical characterization, spectroscopic grade solvents were used throughout all measurements. Absorption spectra were recorded with a Varian Cary 50 Conc spectrophotometer. All *ε* values are given in dm^3^ mol^−1^ cm^−1^.

Dichloromethane was dried and distilled over K_2_CO_3_, and ethanol was dried and distilled over sodium. Pd(OAc)_2_ (Sigma-Aldrich) was used without further purification. *rac*-LA (Sigma-Aldrich) was sublimed under an argon atmosphere repeatedly for further purification and stored in a glove box. All other solvents and reagents were commercially available and used as received. Ligands **L1–L3** were prepared according to literature-reported procedures [44, 45]. Polymerization experiments and characterization of the polymers were performed according to described procedures [12].

### General procedure for the synthesis of **1–3**

The ethanolic solution (10 cm^3^) of the Schiff base (1 mmol) and the palladium acetate (0.5 mmol) in 10 cm^3^ ethanol were mixed thoroughly and the mixture was heated under reflux for 2 h and then cooled to room temperature. After filtration the resulting solution was evaporated to dryness and the residue was recrystallized from dichloromethane.

#### *Bis[2*-*[(4*-*methoxyphenyl)iminomethyl]*-*4,6*-*dimethylphenolato*-κ^*2*^*N,O*^*1*^*]palladium(II)* (**1**, C_32_H_32_N_2_O_4_Pd)

Yield: 0.07 g (79%); ^1^H NMR (300 MHz, CDCl_3_): *δ* = 1.26 (s, C*H*_3_, 6H), 2.12 (s, C*H*_3_, 6H), 3.84 (s, Ar–O–C*H*_3_, 6H), 6.76 (s, *Ar*–H, 2H), 6.83 (s, *Ar*–H, 2H), 6.90-6.93 (d, *J* = 9 Hz, *Ar*–H, 4H), 7.33–7.36 (d, *J* = 9 Hz, *Ar*–H, 4H), 7.59 (s, *CH*=N, 2H) ppm; ^13^C{^1^H} NMR (75 MHz, CDCl_3_): *δ* = 15.77 (*C*H_3_), 20.10 (*C*H_3_), 55.88 (Ar–O–*C*H_3_), 114.26 (*Ar*–C), 114.74 (*Ar*–C), 119.05 (*Ar*–C), 123.60 (*Ar*–C), 125.48 (*Ar*–C), 129.59 (*Ar*–C), 131.23 (*Ar*–C), 137.09 (*Ar*–C), 144.06 (*Ar*–C), 158.37 (*Ar*–O), 163.35 (*C*H=N) ppm; IR (ATR): $\bar{\nu }$ = 1617 (–CH=N), 1506 (Ar-OMe) cm^−1^; MS (ESI): *m*/*z* calculated for C_32_H_32_N_2_O_4_PdNa ([M + Na]^+^) 637.13, found 637.33.

#### *Bis[2,4*-*dibromo*-*6*-*[(2,6*-*diisopropylphenyl)iminomethyl]phenolato*-κ^*2*^*N,O*^*1*^*]palladium(II)* (**2**, C_38_H_40_Br_4_N_2_O_2_Pd)

Yield: 0.07 g (73%); ^1^H NMR (300 MHz, CDCl_3_): *δ* = 1.17–1.20 (d, *J* = 9 Hz, CH(C*H*_3_)_2_, 12H), 1.29–1.32 (d, *J* = 9 Hz, CH(C*H*_3_)_2_, 12H), 3.52–3.66 (m, C*H*(CH_3_)_2_, 4H), 7.15–7.16 (d, *J* = 3 Hz, *Ar*–H, 2H), 7.18-7.19 (d, *J* = 3 Hz, *Ar*–H, 2H), 7.21 (s, *Ar*–H, 3H), 7.28–7.29 (d, *J* = 3 Hz, *Ar*–H, 1H), 7.44 (s, *Ar*–H, 2H), 7.52–7.53 (d, *J* = 3 Hz,C*H* = N, 2H) ppm; ^13^C{^1^H} NMR (75 MHz, CDCl_3_): *δ* = 23.48 (CH(*C*H_3_)_2_), 24.42 (CH(*C*H_3_)_2_), 28.81 (*C*H(CH_3_)_2_), 105.27 (*Ar*–C), 114.32 (*Ar*–C), 120.93 (*Ar*–C), 124.70 (*Ar*–C), 127.54 (*Ar*–C), 135.44 (*Ar*–C), 140.31 (*Ar*–C), 142.02 (*Ar*–C), 145.07 (*Ar*–C), 159.98 (*Ar*–O), 163.25 (*C*H=N) ppm; IR (ATR): $\bar{\nu }$ = 1607(–CH=N) cm^−1^; MS (ESI): *m*/*z* calculated for C_38_H_41_Br_4_N_2_O_2_Pd ([M + H]^+^) 983.780, found 983.497.

#### *Bis[2*-*[(4*-*methoxybenzyl)iminomethyl]*-*4*-*methyl*-*6*-*(tert*-*butyl)phenolato*-κ^*2*^*N,O*^*1*^*]palladium(II)* (**3**, C_40_H_48_N_2_O_4_Pd)

Yield: 0.06 g (75%); ^1^H NMR (300 MHz, CDCl_3_): *δ* = 1.37 (s, C(C*H*_3_)_3_, 18H), 2.18 (s, C*H*_3_, 6H), 3.84 (s, Ar–O–C*H*_3_, 6H), 4.98 (s, Ar–C*H*_2_, 4H), 6.79–6.80 (d, *J* = 3 Hz, *Ar*–H, 2H), 6.86–6.89 (d, *J* = 9 Hz, *Ar*–H, 4H), 7.06–7.07 (d, *J* = 3 Hz, *Ar*–H, 2H), 7.48 (s, *Ar*–H, 2H), 7.53–7.56 (d, *J* = 9 Hz, C*H*=N, 2H) ppm; ^13^C{^1^H} NMR (75 MHz, CDCl_3_): *δ* = 20.40 (*C*H_3_), 29.52 (C(*C*H_3_)_3_), 35.22 (*C*(CH_3_)_3_), 55.38 (Ar–O–*C*H_3_), 60.87 (Ar–*C*H_2_), 114.31 (*Ar*–C), 122.32 (*Ar*–C), 123.14 (*Ar*–C), 129.35 (*Ar*–C), 130.23 (*Ar*–C), 131.16 (*Ar*–C), 131.83 (*Ar*–C), 133.35 (*Ar*–C), 139.33 (*Ar*-C), 159.12 (*Ar*–O), 165.95 (*C*H=N) ppm; IR (ATR): $\bar{\nu }$ = 1624 (–CH=N), 1512 (Ar–OMe) cm^−1^; MS (ESI): *m*/*z* calculated for C_40_H_49_N_2_O_4_Pd ([M + H]^+^) 727.27, found 727.60.

### X-ray structure determination of compounds 1–3

Suitable single crystals for X-ray diffraction were grown from concentrated dichloromethane solution of the respective compounds over a period of 7 days. Single crystal analysis were carried out on a Bruker SMART APEX and a Bruker Smart X2S diffractometer operating with Mo Kα radiation (*λ* = 0.71073 Å). The structures were solved by direct methods (SHELXS-97, SIR-97) [53, 54] and refined by full-matrix least squares on *F* ^2^ (SHELXL-97) [55]. The H atoms were calculated geometrically, and a riding model was applied in the refinement process. These data were deposited with CCDC with the following numbers: CCDC 1585795–1585797. These data can be obtained free of charge from the Cambridge Crystallographic Data Centre at http://www.ccdc.cam.ac.uk. The crystal data are given in Table 4.

**Table 4:** Crystal structure data for **1**–**3**

Complex	**1**	**2**	**3**
Empirical formula	C_32_H_32_N_2_O_4_Pd	C_38_H_40_Br_4_N_2_O_2_Pd	C_40_H_48_N_2_O_4_Pd
Formula weight	615.00	982.76	727.20
Crystal system	Monoclinic	Triclinic	Triclinic
Space group	*P*2_1_/*n*	*P* $\bar{1}$	*P* $\bar{1}$
*a*/Å	12.7329 (12)	9.35 (2)	14.9031 (15)
*b*/Å	9.3575 (10)	9.46 (2)	15.4962 (16)
*c*/Å	12.7447 (12)	12.40 (2)	17.2469 (16)
*α*/°	90	112.25 (2)	72.462 (3)
*β*/°	12.7447 (12)	95.71 (2)	77.170 (3)
*γ*/°	90	99.12 (2)	80.781 (3)
*V*/Å^3^	1382.7 (2)	987 (4)	3684.4 (6)
*Z*	2	1	4
Temp/K	260 (2)	300 (2)	300 (2)
*D*_calc_/g cm^−3^	1.477	1.654	1.311
Reflns collected	24,978	3725	32,284
Unique reflns	2444	1628	12,644
Observ. reflns [*I* ≥ 2σ(*I*)]	1949	1192	7636
Param. refined/restraints	181/0	220/0	868/0
Absorption correction	Multi-scan	Multi-scan	Multi-scan
*T*_*min*_ */T*_*max*_	0.68/0.85	0.61/0.74	0.24/0.89
*R*_1_/*wR*_2_	0.032/0.086	0.056/0.160	0.094/0.237
∆σ_fin_(max/min)/eÅ^−3^	0.29/− 0.71	0.17/− 0.15	1.49/− 1.22
CCDC	1585795	1585796	1585797

*R*_*1*_ = ∑|*F*_0_| − |*F*_c_|/∑|*F*_0_|, *wR*_*2*_ = [∑(*F*_0_^2^ − *F*_c_^2^)^2^/∑*w*(*F*_0_^2^)^2^]^1/2^

## Electronic supplementary material

Below is the link to the electronic supplementary material.

Supplementary material 1 (PDF 122 kb)

Supplementary material 2 (PDF 150 kb)

Supplementary material 3 (PDF 208 kb)

Supplementary material 4 (CIF 14 kb)

Supplementary material 5 (CIF 16 kb)

Supplementary material 6 (CIF 48 kb)

Supplementary material 7 (DOCX 1576 kb)

## References

[1] Cozzi PG (2004). Chem Soc Rev.

[2] Hernández-Molina R, Mederos A (2003) Acyclic and Macrocyclic Schiff Base Ligands. In: McCleverty JA, Meyer TJ (eds) Comprehensive coordination chemistry II, vol 1. Elsevier, p 411

[3] Li XF, Li YS (2002). J Polym Sci Part A Polym Chem.

[4] Chellan P, Stringer T, Shokar A, Dornbush PJ, Vazquez-Anaya G, Land KM, Chibale K, Smith GS (2011). J Inorg Biochem.

[5] Cui J, Zhang M, Zhang Y (2010). Inorg Chem Commun.

[6] Younkin TR, Connor EF, Henderson JI, Friedrich SK, Grubbs RH, Bansleben DA (2000). Science.

[7] Henderson W, Evans C, Nicholson BK, Fawcett J (2003) Dalton Trans:2691

[8] Zheng F, Hutton AT, van Sittert CGCE, Moss JR, Mapolie SF (2013). Dalton Trans.

[9] Chellan P, Shunmoogam-Gounden N, Hendricks DT, Gut J, Rosenthal PJ, Lategan C, Smith PJ, Chibale K, Smith GS (2010) Eur J Inorg Chem:3520

[10] Komiya N, Okada M, Fukumoto K, Jomori D, Naota T (2011). J Am Chem Soc.

[11] Komiya N, Muraoka T, Iida M, Miyanaga M, Takahashi K, Naota T (2011). J Am Chem Soc.

[12] Mandal M, Oppelt K, List M, Teasdale I, Chakraborty D, Monkowius U (2016). Monatsh Chem.

[13] Xu G, Luo Q, Eibauer S, Rausch RF, Stempfhuber S, Zabel M, Yersin H, Reiser O (2011). Dalton Trans.

[14] Chow PK, Cheng G, Tong GSM, Ma C, Kwok WM, Ang WH, Chung CYS, Yang C, Wang F, Che CM (2016). Chem Sci.

[15] Tong GSM, Chow PK, To WP, Kwok WM, Che CM (2014). Chem Eur J.

[16] Williams JAG (2007). Top Curr Chem.

[17] Yersin H, Rausch AF, Czerwieniec R, Hofbeck T, Fischer T (2011). Coord Chem Rev.

[18] Saha TK, Mandal M, Chakraborty D, Ramkumar V (2013). New J Chem.

[19] Mandal M, Monkowius U, Chakraborty D (2016). New J Chem.

[20] Chakraborty D, Chokkapu ER, Mandal M, Gowda RR, Ramkumar V (2016). ChemistrySelect.

[21] Rajashekhar B, Mandal M, Chakraborty D, Ramkumar V (2017). ChemistrySelect.

[22] Mandal M, Monkowius U, Chakraborty D (2016). J Polym Res.

[23] Saha TK, Mandal M, Thunga M, Ramkumar V, Chakraborty D (2013). Dalton Trans.

[24] Ragauskas AJ, Williams CK, Davison BH, Tschaplinski T (2006). Science.

[25] Williams CK, Hillmyer MA (2008). Polym Rev.

[26] Dove AP (2008). Chem Commun.

[27] Nicolas J, Mura S, Brambilla D, Mackiewicz N, Couvreur P (2013). Chem Soc Rev.

[28] Dechy-Cabaret O, Martin-Vaca B, Bourissou D (2004). Chem Rev.

[29] Jerome C, Lecomte P (2008). Adv Drug Delivery Rev.

[30] Mandal M, Chakraborty D (2016). J Polym Sci Part A Polym Chem.

[31] Mandal M, Chakraborty D, Ramkumar V (2015). RSC Adv.

[32] Tsai C-Y, Du H-C, Chang J-C, Huang B-H, Ko B-T, Lin C-C (2014). RSC Adv.

[33] Wang L, Poirier V, Ghiotto F, Bochmann M, Cannon RD, Carpentier J-F, Sarazin Y (2014). Macromolecules.

[34] Aluthge DC, Patrick BO, Mehrkhodavandi P (2013). Chem Commun.

[35] Sauer A, Kapelski A, Fliedel C, Dagorne S, Kol M, Okuda J (2013). Dalton Trans.

[36] Dagorne S, Normand M, Kirillov E, Carpentier J-F (2013). Coord Chem Rev.

[37] Nie K, Gu W, Yao Y, Zhang Y, Shen Q (2013). Organometallics.

[38] Joshi H, Prakash O, Sharma AK, Sharma KN, Singh AK (2015) Eur J Inorg Chem:1542

[39] Blackburn OA, Coe BJ, Fielden J, Helliwell M, McDouall JJW, Hutchings MG (2010). Inorg Chem.

[40] Roy S, Saha R, Mondal TK, Sinha C (2014). Inorg Chim Acta.

[41] Feng ZQ, Yang XL, Ye YF, Hao LY (2014). Bull Korean Chem Soc.

[42] Khorshidifard M, Rudbari HA, Askari B, Sahihi M, Farsani MR, Jalilian F, Bruno G (2015). Polyhedron.

[43] Tajuddin AM, Bahron H, Zaki HM, Kassim K, Chantrapromma S (2015). Acta Crystallogr.

[44] Kasumov VT, Köksal F, Sezer A (2005). Polyhedron.

[45] Bhunora S, Mugo J, Bhaw-Luximon A, Mapolie S, Wyk JV, Darkwa J, Nordlander E (2011). Appl Organomet Chem.

[46] Swierczynski D, Luboradzki R, Dolgonos G, Lipkowski J, Schneider H-J (2005) Eur J Org Chem:1172

[47] Nagels N, Hauchecorne D, Herrebout WA (2013). Molecules.

[48] Masutani K, Yoshiharu K (2015) PLA Synthesis and Polymerization. In: Jiménez A, Peltzer M, Ruseckaite R (eds), Poly(lactic acid) science and technology: processing, properties, additives and applications. RSC polymer chemistry series no. 12, The Royal Society of Chemistry, p 3

[49] Kricheldorf HR, Mang T, Jonte JM (1984). Macromolecules.

[50] Dubois P, Jacobs C, Jérôme R, Teyssié P (1991). Macromolecules.

[51] Stevels WM, Ankone MJ, Dijkstra PJ, Feijen J (1996). Macromolecules.

[52] Chamberlain BM, Jazdzewski BA, Pink M, Hillmyer MA, Tolman WB (2000). Macromolecules.

[53] Sheldrick GM (1997). SHELXS-97, program for the solution of crystal structures. Göttingen, Germany. See also: Sheldrick GM (1990). Acta Crystallogr.

[54] Altomare A, Burla MC, Camalli M, Cascarano GL, Giacovazzo C, Guagliardi A, Moliterni AGG, Polidori G, Spagna R (1999). J Appl Cryst.

[55] Sheldrick GM (1997). SHELXL-97, program for crystal structure refinement. Göttingen, Germany. See also Sheldrick GM (2008). Acta Crystallogr.

