# Supplementary material for: Palladium complexes containing imino phenoxide ligands: synthesis, luminescence, and their use as catalysts for the ring-opening polymerization of rac-lactide
Source: Monatsh Chem. 2017 Dec 12;149(4):783–90. doi: 10.1007/s00706-017-2119-1 (PMC5906497; doi:10.1007/s00706-017-2119-1)

checkCIF/PLATON report

You have not supplied any structure factors. As a result the full set of tests cannot be run.

THIS REPORT IS FOR GUIDANCE ONLY. IF USED AS PART OF A REVIEW PROCEDURE FOR PUBLICATION, IT SHOULD NOT REPLACE THE EXPERTISE OF AN EXPERIENCED CRYSTALLOGRAPHIC REFEREE.

No syntax errors found.   CIF dictionary   Interpreting this report

Datablock: I

```
Bond precision:  C-C = 0.0046 A          Wavelength=0.71073

Cell:           a=12.7329(12)    b=9.3575(10)       c=12.7447(12)
                alpha=90         beta=114.414(3)    gamma=90
Temperature:    260 K

                Calculated                 Reported
Volume          1382.7(2)                  1382.7(2)
Space group     P 21/n                     P 1 21/n 1
Hall group      -P 2yn                     -P 2yn
Moiety formula  C32 H32 N2 O4 Pd           ?
Sum formula     C32 H32 N2 O4 Pd           C32 H32 N2 O4 Pd
Mr              615.00                     615.00
Dx,g cm-3       1.477                      1.477
Z               2                          2
Mu (mm-1)       0.711                      0.711
F000            632.0                      632.0
F000'           630.25
h,k,lmax        15,11,15                   15,11,15
Nref            2458                       2444
Tmin,Tmax       0.815,0.843                0.680,0.850
Tmin'           0.657

Correction method= # Reported T Limits: Tmin=0.680 Tmax=0.850
AbsCorr = MULTI-SCAN

Data completeness= 0.994          Theta(max)= 25.100

R(reflections)= 0.0324( 1949)     wR2(reflections)= 0.0856( 2444)

S = 1.035                 Npar= 181
```

The following ALERTS were generated. Each ALERT has the format
test-name_ALERT_alert-type_alert-level.
Click on the hyperlinks for more details of the test.

Alert level C

```
ABSTY02_ALERT_1_C  An _exptl_absorpt_correction_type has been given without
            a literature citation. This should be contained in the
            _exptl_absorpt_process_details field.
            Absorption correction given as multi-scan
CRYSC01_ALERT_1_C The word below has not been recognised as a standard
            identifier.
            bloc
CRYSC01_ALERT_1_C No recognised colour has been given for crystal colour.
```

Alert level G

```
PLAT005_ALERT_5_G No Embedded Refinement Details found  in the CIF     Please Do !
```

```
   0 ALERT level A = Most likely a serious problem - resolve or explain
   0 ALERT level B = A potentially serious problem, consider carefully
   3 ALERT level C = Check. Ensure it is not caused by an omission or oversight
   1 ALERT level G = General information/check it is not something unexpected

   3 ALERT type 1 CIF construction/syntax error, inconsistent or missing data
   0 ALERT type 2 Indicator that the structure model may be wrong or deficient
   0 ALERT type 3 Indicator that the structure quality may be low
   0 ALERT type 4 Improvement, methodology, query or suggestion
   1 ALERT type 5 Informative message, check
```

It is advisable to attempt to resolve as many as possible of the alerts in all categories. Often the minor alerts point to easily fixed oversights, errors and omissions in your CIF or refinement strategy, so attention to these fine details can be worthwhile. In order to resolve some of the more serious problems it may be necessary to carry out additional measurements or structure refinements. However, the purpose of your study may justify the reported deviations and the more serious of these should normally be commented upon in the discussion or experimental section of a paper or in the "special_details" fields of the CIF. checkCIF was carefully designed to identify outliers and unusual parameters, but every test has its limitations and alerts that are not important in a particular case may appear. Conversely, the absence of alerts does not guarantee there are no aspects of the results needing attention. It is up to the individual to critically assess their own results and, if necessary, seek expert advice.

Publication of your CIF in IUCr journals

A basic structural check has been run on your CIF. These basic checks will be run on all CIFs submitted for publication in IUCr journals (*Acta Crystallographica*, *Journal of Applied Crystallography*, *Journal of Synchrotron Radiation*); however, if you intend to submit to *Acta Crystallographica Section C* or *E* or *IUCrData*, you should make sure that full publication checks are run on the final version of your CIF prior to submission.

Publication of your CIF in other journals

Please refer to the *Notes for Authors* of the relevant journal for any special instructions relating to CIF submission.

PLATON version of 13/08/2017; check.def file version of 27/07/2017

Datablock I - ellipsoid plot

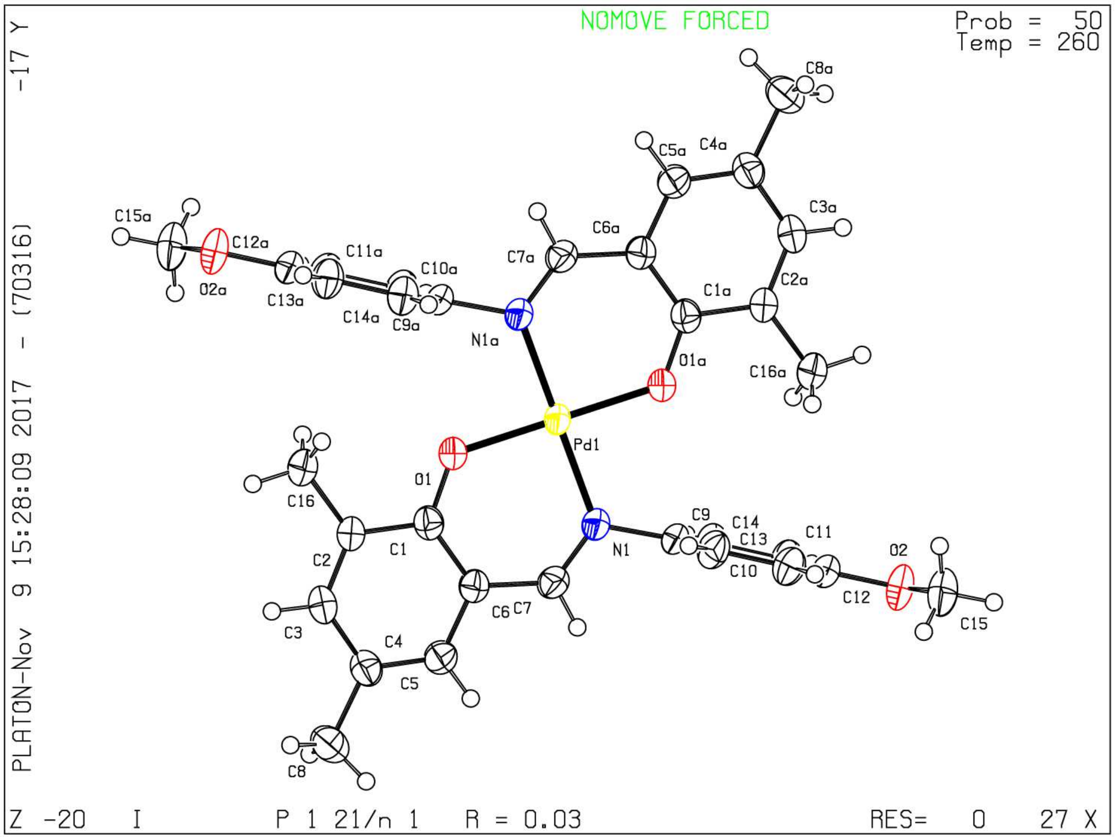

Supplement: Supplementary file 1 — Supplementary material 1 (PDF 122 kb) [file 706_2017_2119_MOESM1_ESM.pdf]
