# Supplementary material for: Palladium complexes containing imino phenoxide ligands: synthesis, luminescence, and their use as catalysts for the ring-opening polymerization of rac-lactide
Source: Monatsh Chem. 2017 Dec 12;149(4):783–90. doi: 10.1007/s00706-017-2119-1 (PMC5906497; doi:10.1007/s00706-017-2119-1)

checkCIF/PLATON report

You have not supplied any structure factors. As a result the full set of tests cannot be run.

THIS REPORT IS FOR GUIDANCE ONLY. IF USED AS PART OF A REVIEW PROCEDURE FOR PUBLICATION, IT SHOULD NOT REPLACE THE EXPERTISE OF AN EXPERIENCED CRYSTALLOGRAPHIC REFEREE.

No syntax errors found. CIF dictionary Interpreting this report

Datablock: I

```
Bond precision:  C-C = 0.0127 A          Wavelength=0.71073

Cell:           a=14.9031(15)      b=15.4962(16)     c=17.2469(16)
                alpha=72.462(3)    beta=77.170(3)    gamma=80.781(3)
Temperature:    300 K

                 Calculated                 Reported
Volume           3684.4(6)                  3684.4(6)
Space group      P -1                       P -1
Hall group       -P 1                       -P 1
Moiety formula   C40 H48 N2 O4 Pd           ?
Sum formula      C40 H48 N2 O4 Pd           C40 H48 N2 O4 Pd
Mr               727.20                     727.20
Dx,g cm-3        1.311                      1.311
Z                4                          4
Mu (mm-1)        0.545                      0.545
F000             1520.0                     1520.0
F000'            1516.57
h,k,lmax         17,18,20                   17,18,20
Nref             13185                      12644
Tmin,Tmax        0.827,0.892                0.240,0.890
Tmin'            0.826

Correction method= # Reported T Limits: Tmin=0.240 Tmax=0.890
AbsCorr = MULTI-SCAN

Data completeness= 0.959          Theta(max)= 25.140

R(reflections)= 0.0942( 7636)     wR2(reflections)= 0.2370( 12644)

S = 0.990                  Npar= 868
```

The following ALERTS were generated. Each ALERT has the format
test-name_ALERT_alert-type_alert-level.
Click on the hyperlinks for more details of the test.

Alert level A

```
REFI020_ALERT_1_A  _refine_ls_extinction_coef is missing
            Extinction coefficient applied in corrections.
SHFSU01_ALERT_2_A  The absolute value of parameter shift to su ratio > 0.20
            Absolute value of the parameter shift to su ratio given   2.506
            Additional refinement cycles may be required.
```

Author Response: All measured crystals were of low quality. Therefore, not better data could be obtained. Additional refinement cycles do not improve the results.

```
PLAT080_ALERT_2_A Maximum Shift/Error ............................       2.51 Why ?
```

Author Response: see above

Alert level B

```
PLAT029_ALERT_3_B _diffrn_measured_fraction_theta_full value Low .      0.959 Why?
PLAT234_ALERT_4_B Large Hirshfeld Difference O3     -- C19          0.30 Ang.
```

Alert level C

```
PLAT048_ALERT_1_C MoietyFormula Not Given (or Incomplete) ........     Please Check
PLAT220_ALERT_2_C Non-Solvent Resd  C   Ueq(max)/Ueq(min) Range         4.6 Ratio
PLAT220_ALERT_2_C Non-Solvent Resd  O   Ueq(max)/Ueq(min) Range         3.5 Ratio
PLAT220_ALERT_2_C Non-Solvent Resd  C   Ueq(max)/Ueq(min) Range         3.8 Ratio
PLAT222_ALERT_3_C Non-Solv.  Resd  H   Uiso(max)/Uiso(min) Range       4.6 Ratio
PLAT222_ALERT_3_C Non-Solv.  Resd  H   Uiso(max)/Uiso(min) Range       4.2 Ratio
PLAT234_ALERT_4_C Large Hirshfeld Difference C16    -- C17          0.16 Ang.
PLAT234_ALERT_4_C Large Hirshfeld Difference C48    -- C50          0.16 Ang.
PLAT241_ALERT_2_C High   'MainMol' Ueq as Compared to Neighbors of       O3 Check
PLAT241_ALERT_2_C High   'MainMol' Ueq as Compared to Neighbors of      C14 Check
PLAT242_ALERT_2_C Low    'MainMol' Ueq as Compared to Neighbors of      C12 Check
PLAT242_ALERT_2_C Low    'MainMol' Ueq as Compared to Neighbors of      C80 Check
PLAT342_ALERT_3_C Low Bond Precision on  C-C Bonds ...............    0.01274 Ang.
PLAT412_ALERT_2_C Short Intra XH3 .. XHn   H19C   ..H37     ..      1.85 Ang.
```

Alert level G

```
PLAT005_ALERT_5_G No Embedded Refinement Details Found  in the CIF     Please Do !
PLAT154_ALERT_1_G The s.u.'s on the Cell Angles are Equal ..(Note)      0.003 Degree
PLAT710_ALERT_4_G Delete 1-2-3 or 2-3-4 Linear Torsion Angle ... #        3 Do !
            N1  -PD1 -N2  -C26  -103.00  3.00   1.555   1.555   1.555   1.555
PLAT710_ALERT_4_G Delete 1-2-3 or 2-3-4 Linear Torsion Angle ... #        6 Do !
            N1  -PD1 -N2  -C32    67.00  3.00   1.555   1.555   1.555   1.555
PLAT710_ALERT_4_G Delete 1-2-3 or 2-3-4 Linear Torsion Angle ... #        7 Do !
            O1  -PD1 -O2  -C20   -54.80  1.90   1.555   1.555   1.555   1.555
PLAT710_ALERT_4_G Delete 1-2-3 or 2-3-4 Linear Torsion Angle ... #       10 Do !
            O2  -PD1 -O1  -C1    -69.00  2.00   1.555   1.555   1.555   1.555
PLAT710_ALERT_4_G Delete 1-2-3 or 2-3-4 Linear Torsion Angle ... #       15 Do !
            N2  -PD1 -N1  -C11  -116.00  3.00   1.555   1.555   1.555   1.555
PLAT710_ALERT_4_G Delete 1-2-3 or 2-3-4 Linear Torsion Angle ... #       18 Do !
```

```
            N2  -PD1 -N1  -C83    59.00  3.00   1.555   1.555   1.555   1.555
PLAT710_ALERT_4_G Delete 1-2-3 or 2-3-4 Linear Torsion Angle ... #       19 Do !
            O8  -PD2 -O6  -C40    69.70  1.50   1.555   1.555   1.555   1.555
PLAT710_ALERT_4_G Delete 1-2-3 or 2-3-4 Linear Torsion Angle ... #       23 Do !
            O6  -PD2 -O8  -C59    56.00  1.50   1.555   1.555   1.555   1.555
PLAT710_ALERT_4_G Delete 1-2-3 or 2-3-4 Linear Torsion Angle ... #       26 Do !
            N4  -PD2 -N3  -C46   102.00  3.00   1.555   1.555   1.555   1.555
PLAT710_ALERT_4_G Delete 1-2-3 or 2-3-4 Linear Torsion Angle ... #       29 Do !
            N4  -PD2 -N3  -C52   -72.00  3.00   1.555   1.555   1.555   1.555
PLAT710_ALERT_4_G Delete 1-2-3 or 2-3-4 Linear Torsion Angle ... #      165 Do !
            N3  -PD2 -N4  -C70   118.00  3.00   1.555   1.555   1.555   1.555
PLAT710_ALERT_4_G Delete 1-2-3 or 2-3-4 Linear Torsion Angle ... #      168 Do !
            N3  -PD2 -N4  -C71   -58.00  3.00   1.555   1.555   1.555   1.555
```

---

3 **ALERT level A** = Most likely a serious problem - resolve or explain
2 **ALERT level B** = A potentially serious problem, consider carefully
14 **ALERT level C** = Check. Ensure it is not caused by an omission or oversight
14 **ALERT level G** = General information/check it is not something unexpected

3 ALERT type 1 CIF construction/syntax error, inconsistent or missing data
10 ALERT type 2 Indicator that the structure model may be wrong or deficient
4 ALERT type 3 Indicator that the structure quality may be low
15 ALERT type 4 Improvement, methodology, query or suggestion
1 ALERT type 5 Informative message, check

---

checkCIF publication errors

Alert level A

PUBL003_ALERT_1_A The contact author’s name is missing,
            _publ_contact_author_name.
PUBL005_ALERT_1_A _publ_contact_author_email, _publ_contact_author_fax and
            _publ_contact_author_phone are all missing.
            At least one of these should be present.
PUBL006_ALERT_1_A _publ_requested_journal is missing
            e.g. ’Acta Crystallographica Section C’
PUBL009_ALERT_1_A _publ_author_name is missing. List of author(s) name(s).
PUBL010_ALERT_1_A _publ_author_address is missing. Author(s) address(es).

---

5 **ALERT level A** = Data missing that is essential or data in wrong format
0 **ALERT level G** = General alerts. Data that may be required is missing

---

Publication of your CIF

You should attempt to resolve as many as possible of the alerts in all categories. Often the minor alerts point to easily fixed oversights, errors and omissions in your CIF or refinement strategy, so attention to these fine details can be worthwhile. In order to resolve some of the more serious problems it may be necessary to carry out additional measurements or structure refinements. However, the nature of your study may justify the reported deviations from journal submission requirements and the more serious of these should be commented upon in the discussion or experimental section of a paper or in the "special_details" fields of the CIF. *checkCIF* was carefully designed to identify outliers and unusual parameters, but every test has its limitations and alerts that are not important in a particular case may appear. Conversely, the absence of alerts does not guarantee there are no aspects of the results needing attention. It is up to the individual to critically assess their own results and, if necessary, seek expert advice.

If level A alerts remain, which you believe to be justified deviations, and you intend to submit this CIF for publication in a journal, you should additionally insert an explanation in your CIF using the Validation Reply Form (VRF) below. This will allow your explanation to be considered as part of the review process.

Validation response form

Please find below a validation response form (VRF) that can be filled in and pasted into your CIF.

```
# start Validation Reply Form
_vrf_PUBL003_GLOBAL
;
PROBLEM: The contact author’s name is missing,
RESPONSE: ...
;
_vrf_PUBL005_GLOBAL
;
PROBLEM: _publ_contact_author_email, _publ_contact_author_fax and
RESPONSE: ...
;
_vrf_PUBL006_GLOBAL
;
PROBLEM: _publ_requested_journal is missing
RESPONSE: ...
;
_vrf_PUBL009_GLOBAL
;
PROBLEM: _publ_author_name is missing. List of author(s) name(s).
RESPONSE: ...
;
_vrf_PUBL010_GLOBAL
;
PROBLEM: _publ_author_address is missing. Author(s) address(es).
RESPONSE: ...
;
_vrf_REFI020_I
;
PROBLEM: _refine_ls_extinction_coef is missing
RESPONSE: ...
;
# end Validation Reply Form
```

If you wish to submit your CIF for publication in Acta Crystallographica Section C or E, you should upload your CIF via the web. If you wish to submit your CIF for publication in IUCrData you should upload your CIF via the web. If your CIF is to form part of a submission to another IUCr journal, you will be asked, either during electronic submission or by the Co-editor handling your paper, to upload your CIF via our web site.

PLATON version of 13/08/2017; check.def file version of 08/11/2017

Datablock I - ellipsoid plot

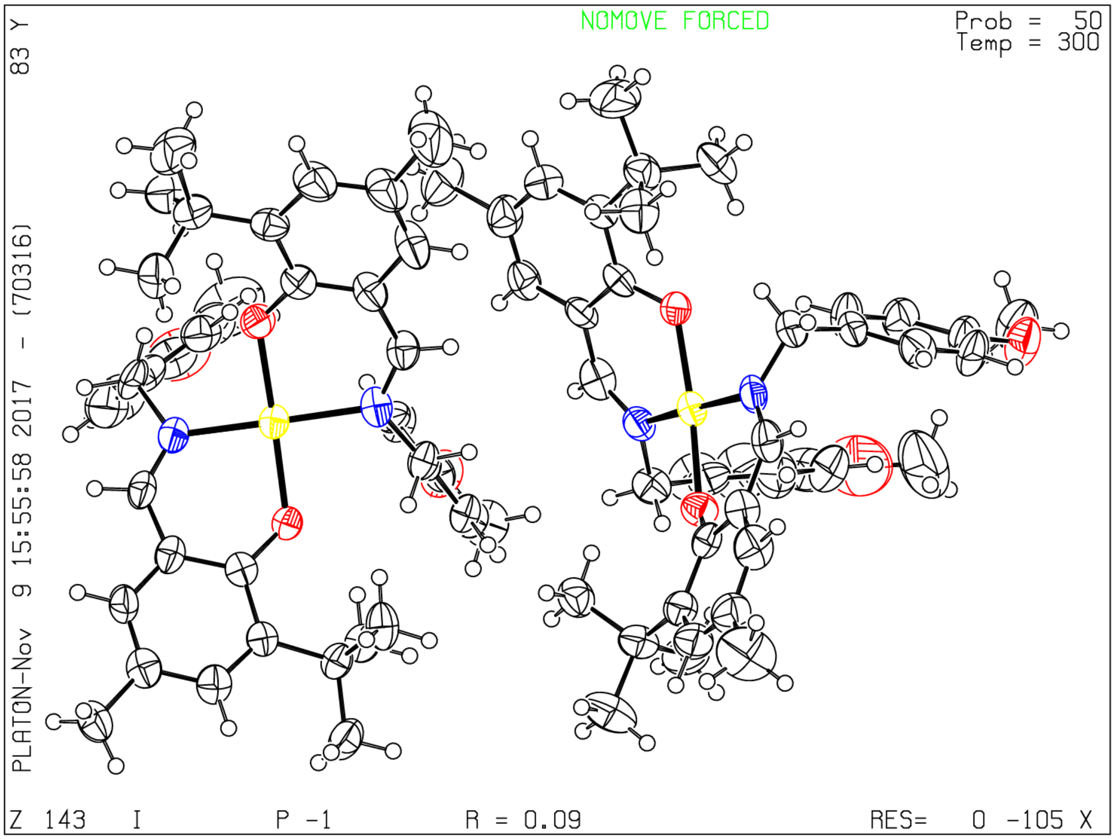

Supplement: Supplementary file 3 — Supplementary material 3 (PDF 208 kb) [file 706_2017_2119_MOESM3_ESM.pdf]
