# Supplementary material for: Palladium complexes containing imino phenoxide ligands: synthesis, luminescence, and their use as catalysts for the ring-opening polymerization of rac-lactide
Source: Monatsh Chem. 2017 Dec 12;149(4):783–90. doi: 10.1007/s00706-017-2119-1 (PMC5906497; doi:10.1007/s00706-017-2119-1)
Supplement: Supplementary file 7 — Supplementary material 7 (DOCX 1576 kb) [file 706_2017_2119_MOESM7_ESM.docx]

**Supporting Information**

Palladium complexes containing imino phenoxide ligands: Synthesis, luminescence and their use as catalysts for the ring-opening polymerization of *rac*-lactide


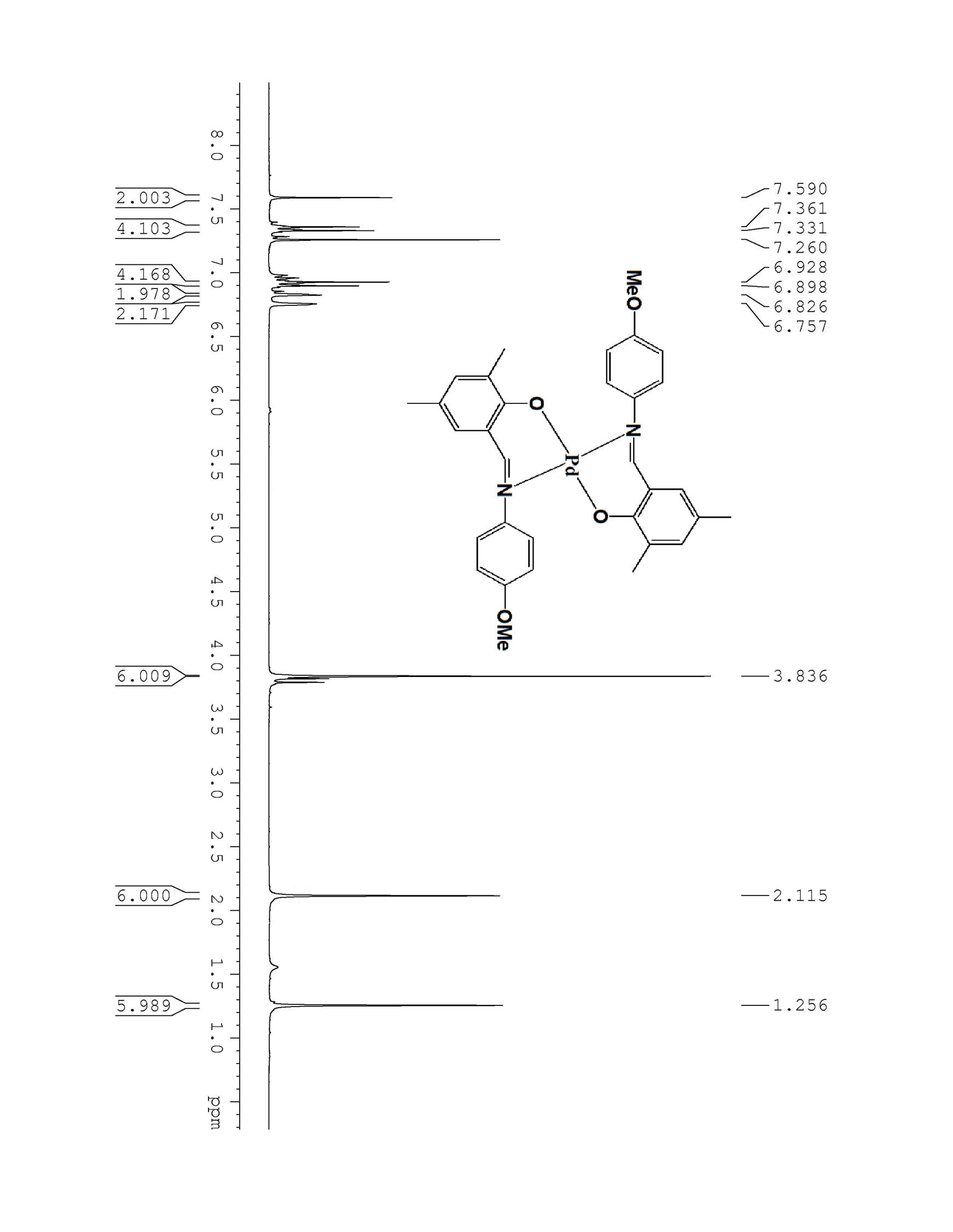


**Fig. S1.** ^1^H NMR spectrum (300 MHz, CDCl_3_) of complex **1**


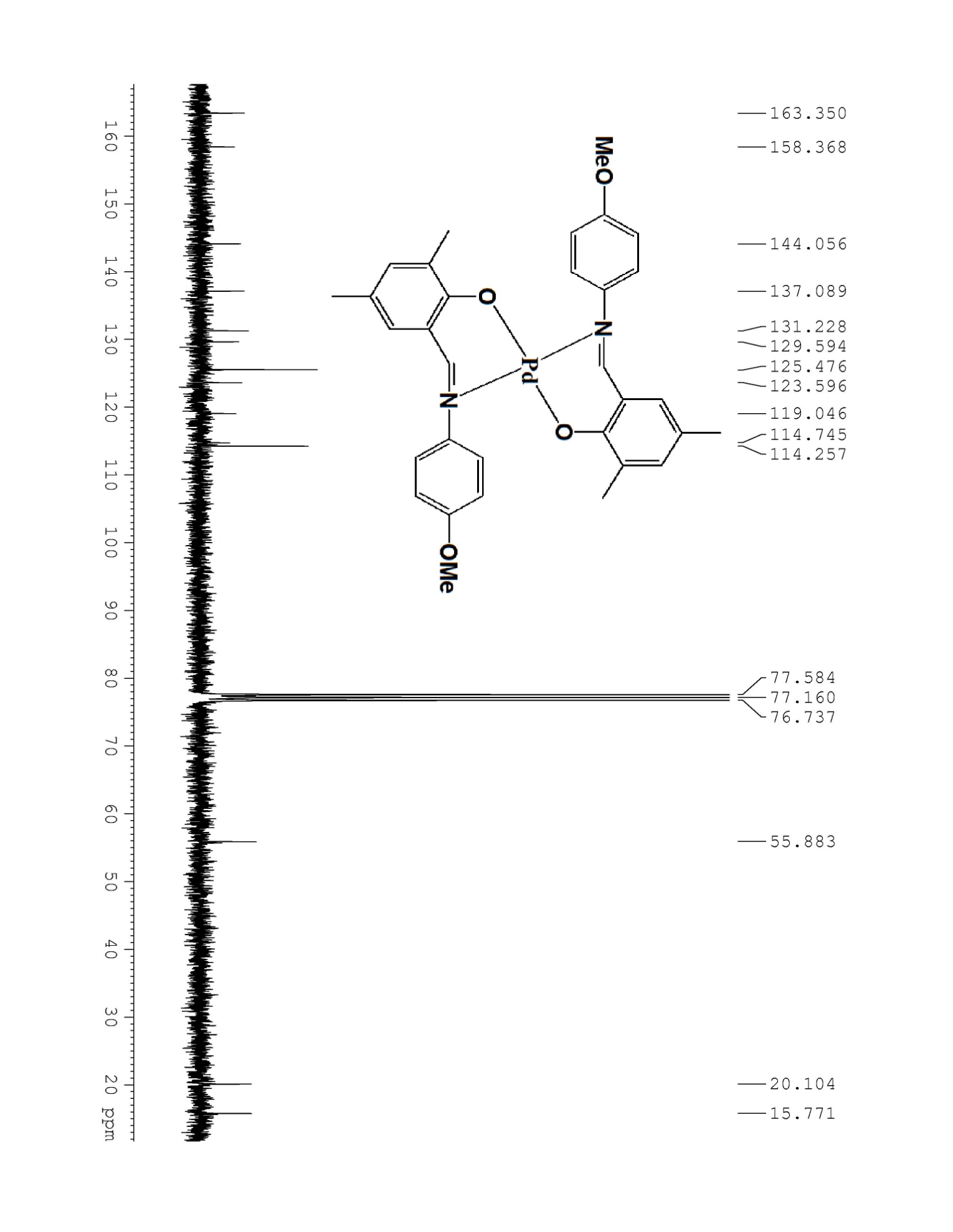


**Fig. S2.** ^13^C{^1^H} NMR spectrum (75 MHz, CDCl_3_) of complex **1**

**Fig. S3.** ESI-mass spectrum of complex **1**


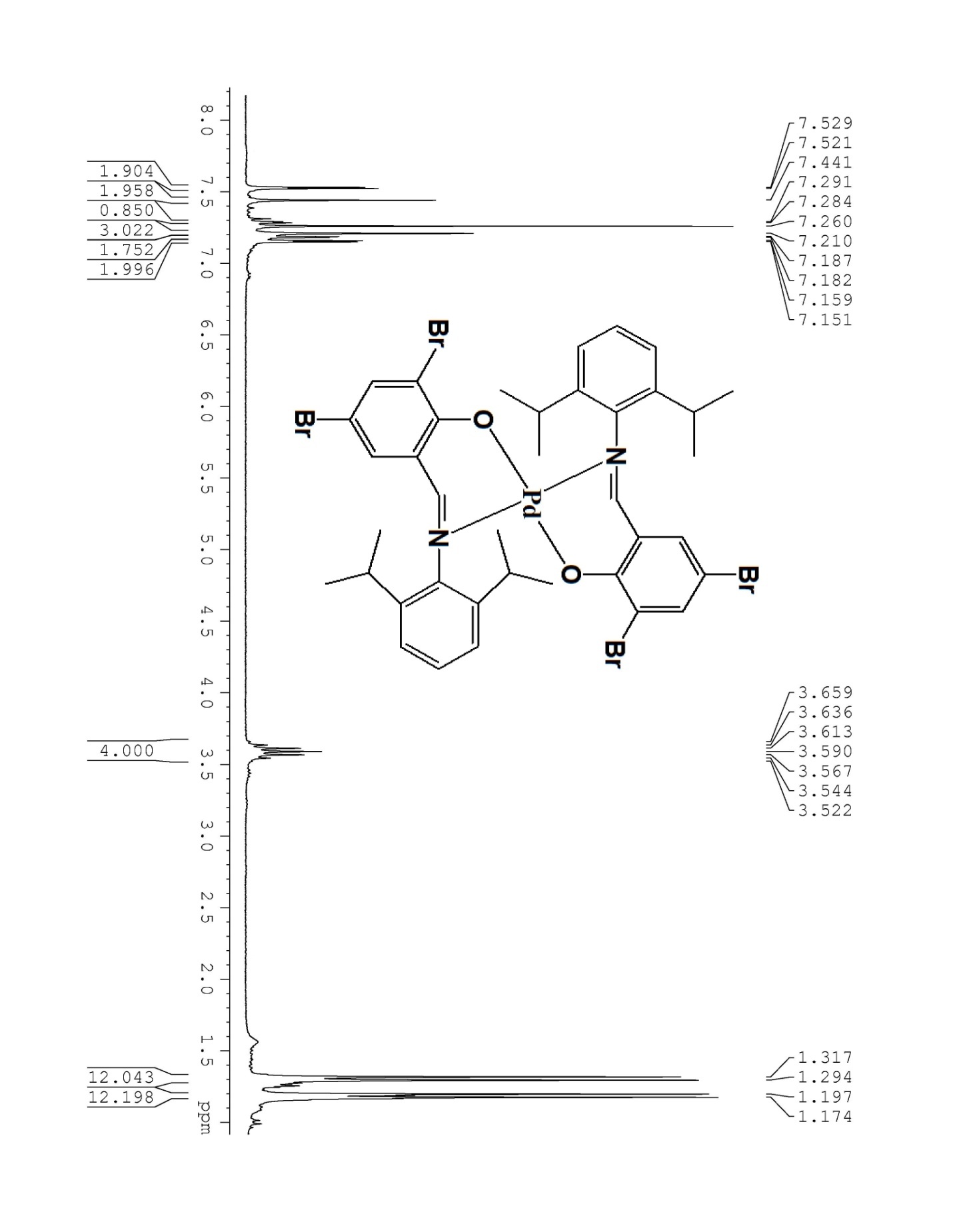


**Fig. S4.** ^1^H NMR spectrum (300 MHz, CDCl_3_) of complex **2**


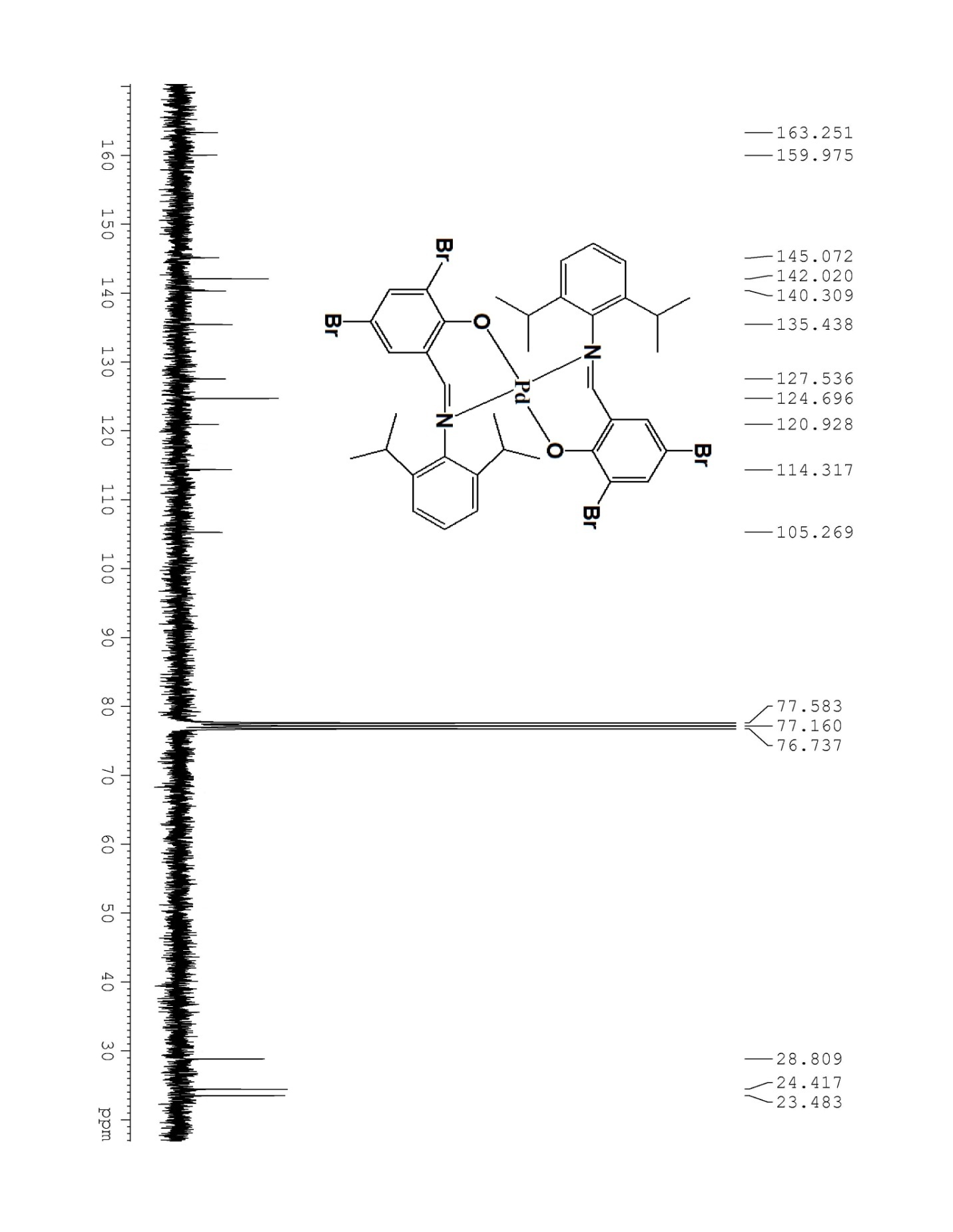


**Fig. S5.** ^13^C{^1^H} NMR spectrum (75 MHz, CDCl_3_) of complex **2**

**Fig. S6.** ESI-mass spectrum of complex **2**


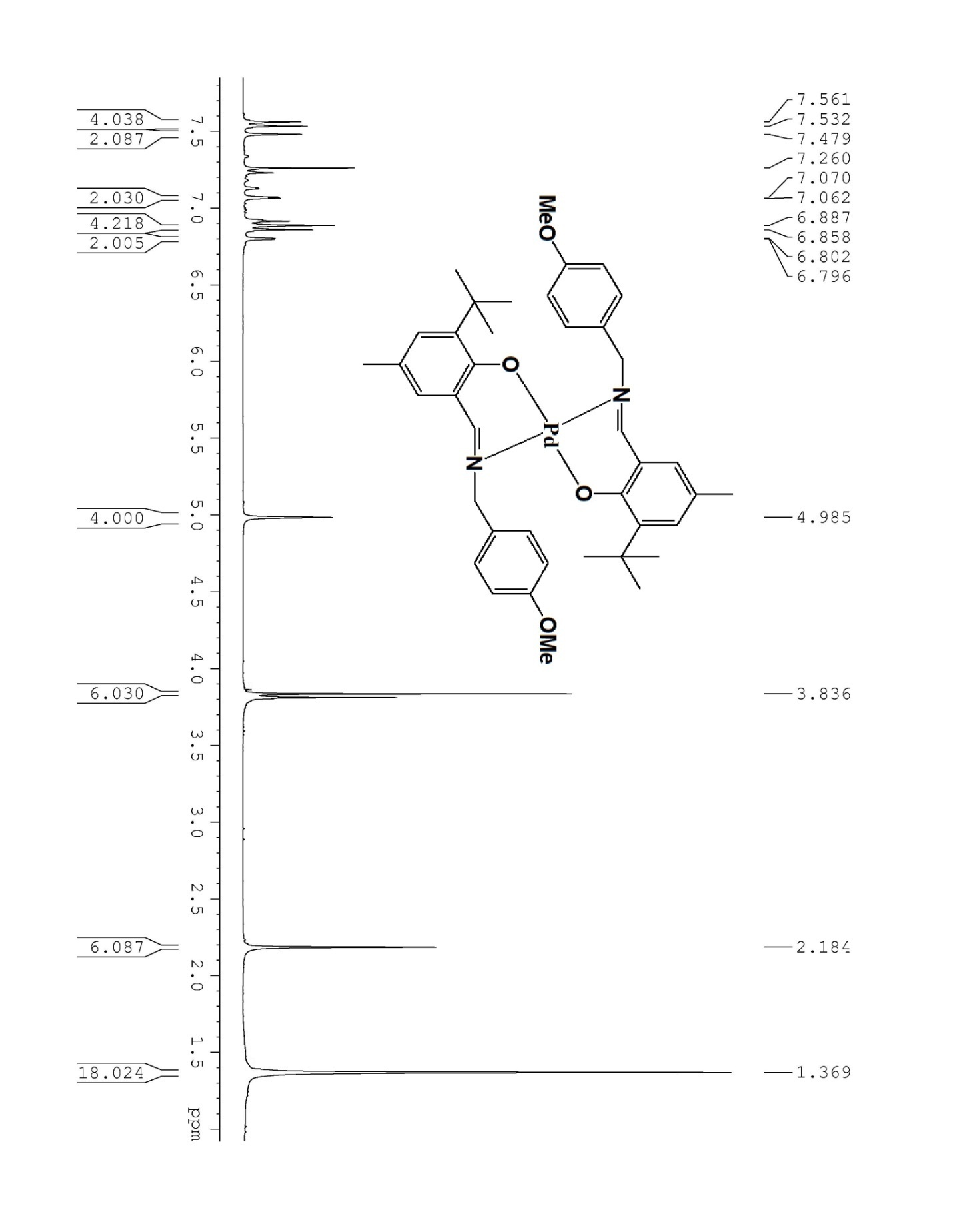


**Fig. S7.** ^1^H NMR spectrum (300 MHz, CDCl_3_) of complex **3**


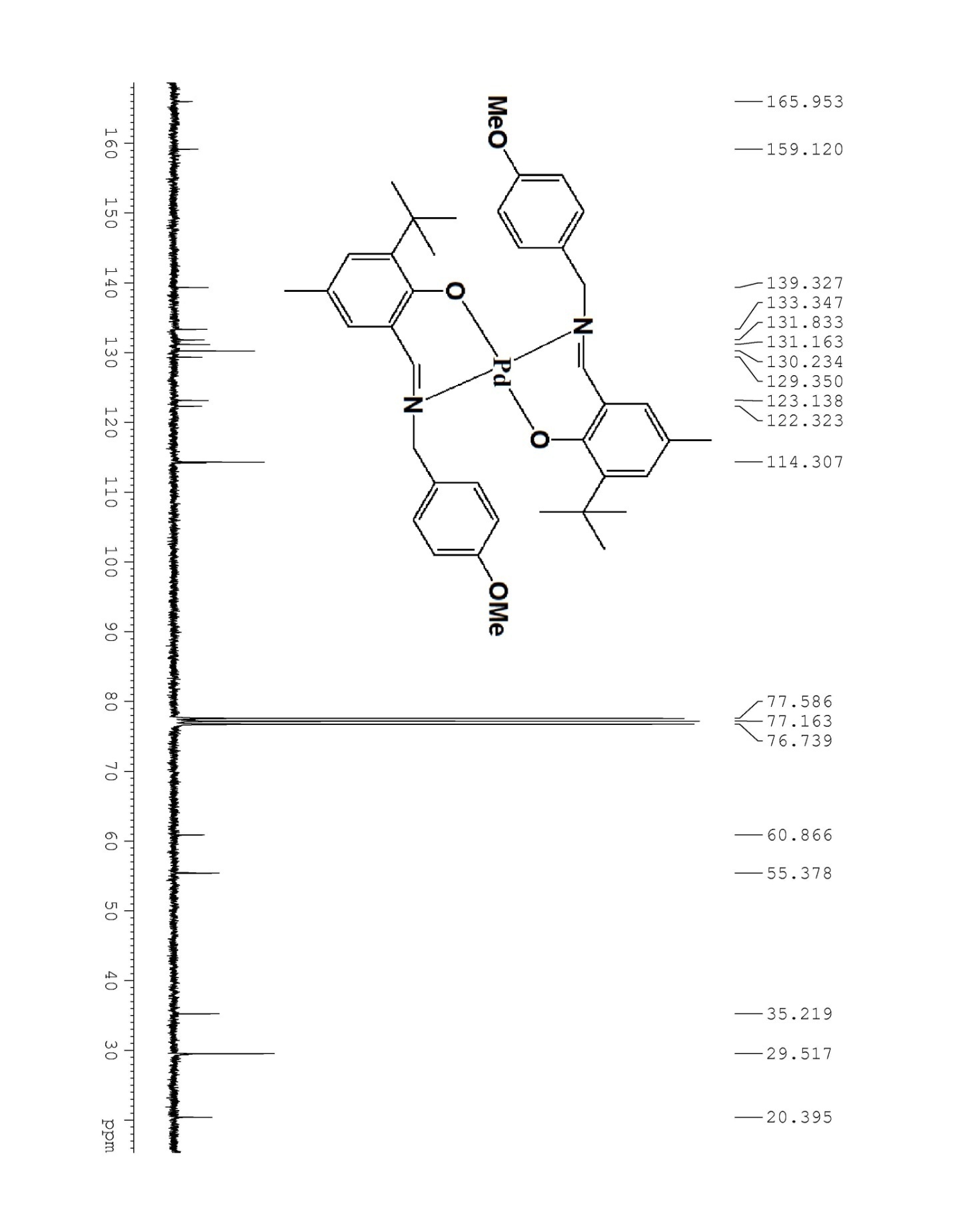


**Fig. S8.** ^13^C{^1^H} NMR spectrum (75 MHz, CDCl_3_) of complex **3**

**Fig. S9.** ESI-mass spectrum of complex **3**


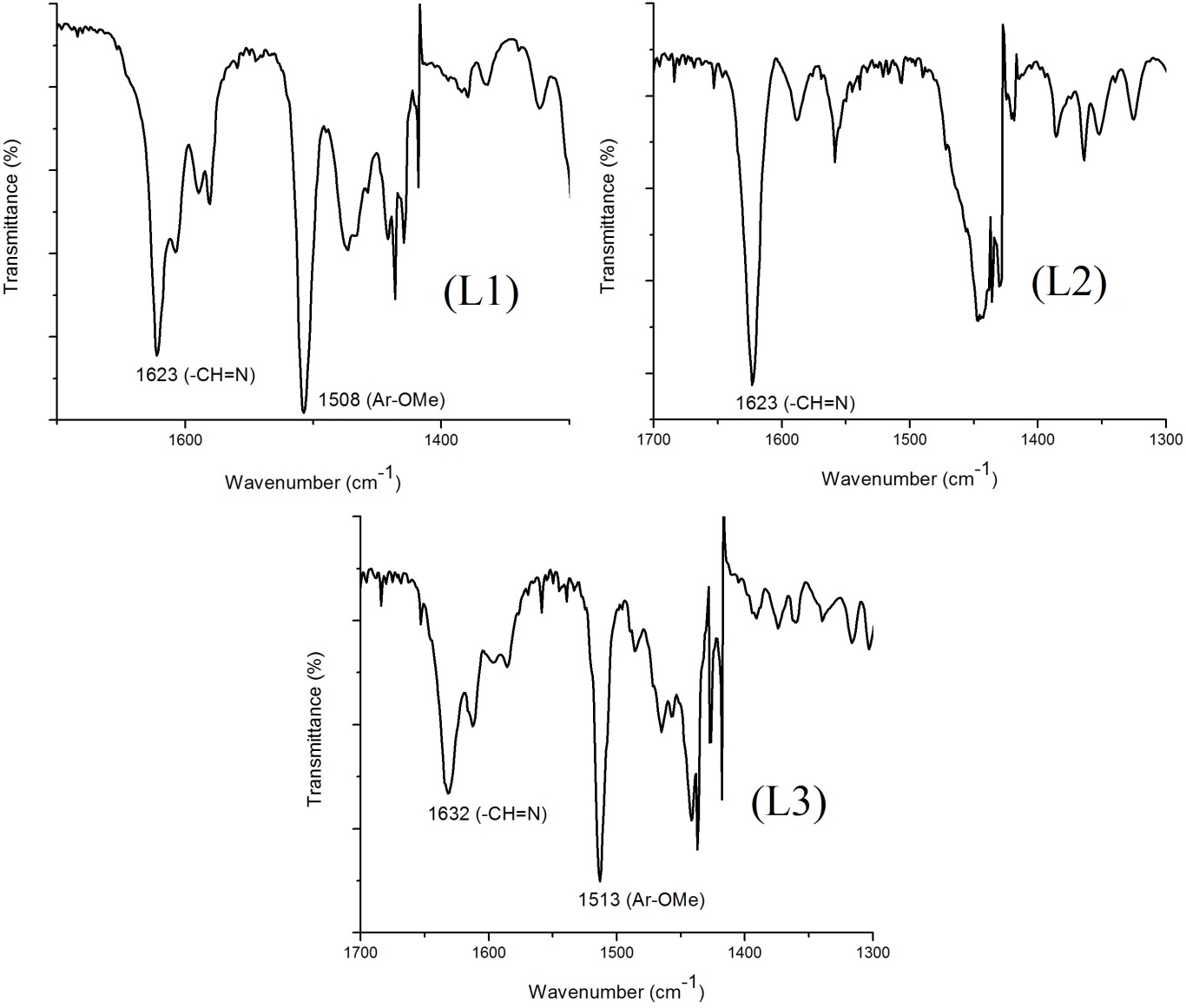


**Fig. S10.** FT-IR spectra of ligands **L1**–**L3**


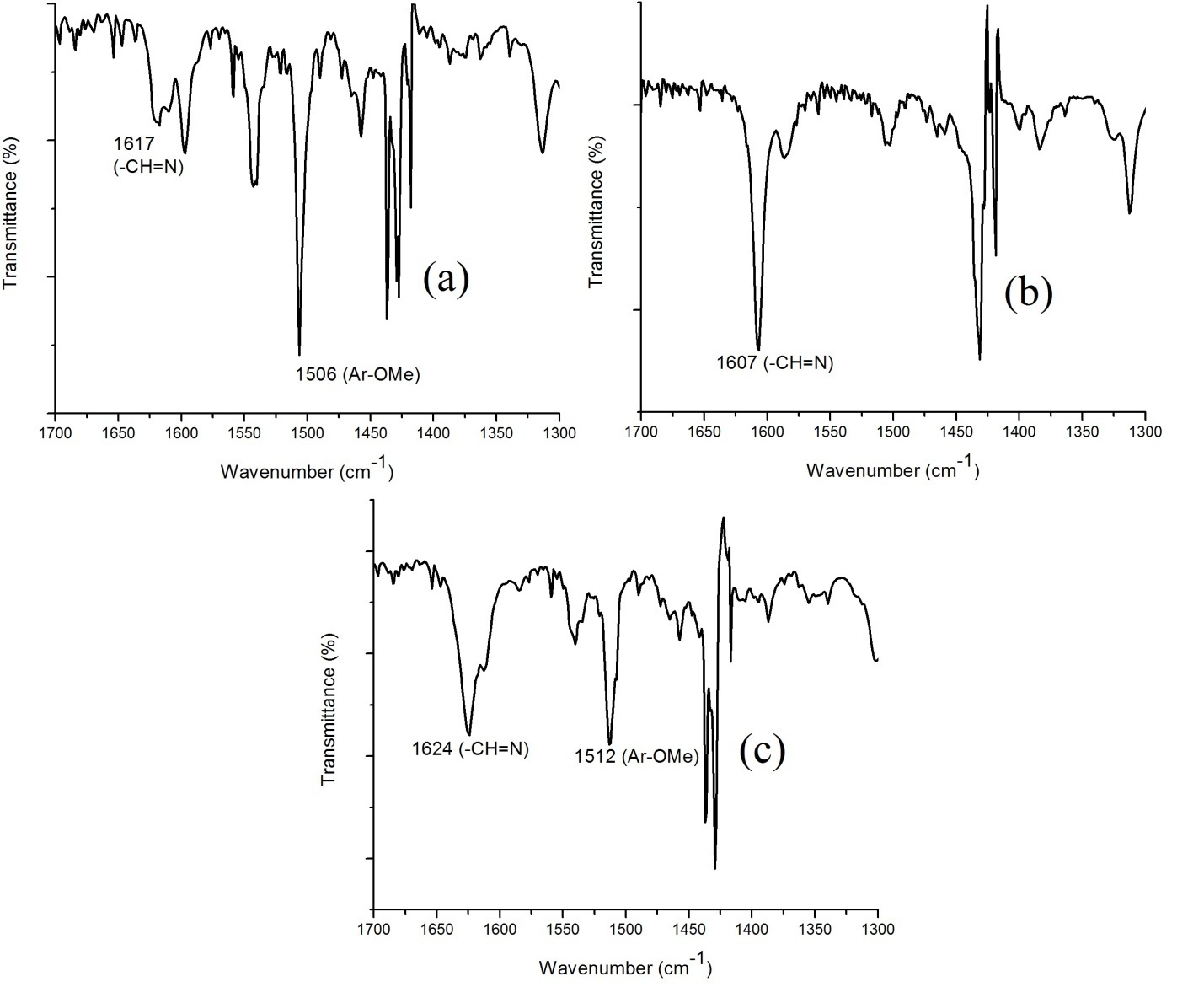


**Fig. S11.** FT-IR spectra of (a) complex **1**, (b) complex **2**, and (c) complex **3**
