# Supplementary material for: Palladium complexes containing imino phenoxide ligands: synthesis, luminescence, and their use as catalysts for the ring-opening polymerization of rac-lactide
Source: Monatsh Chem. 2017 Dec 12;149(4):783–90. doi: 10.1007/s00706-017-2119-1 (PMC5906497; doi:10.1007/s00706-017-2119-1)

checkCIF/PLATON report

Structure factors have been supplied for datablock(s) I

THIS REPORT IS FOR GUIDANCE ONLY. IF USED AS PART OF A REVIEW PROCEDURE FOR PUBLICATION, IT SHOULD NOT REPLACE THE EXPERTISE OF AN EXPERIENCED CRYSTALLOGRAPHIC REFEREE.

No syntax errors found. CIF dictionary Interpreting this report

Datablock: I

```
Bond precision:  C-C = 0.0253 A          Wavelength=0.71073

Cell:           a=9.35(2)          b=9.46(2)        c=12.40(3)
                alpha=112.25(2)    beta=95.71(2)    gamma=99.12(2)
Temperature:    293 K

                Calculated                Reported
Volume          987(4)                    987(4)
Space group     P -1                      P -1
Hall group      -P 1                      -P 1
Moiety formula  C38 H40 Br4 N2 O2 Pd      ?
Sum formula     C38 H40 Br4 N2 O2 Pd      C38 H40 Br4 N2 O2 Pd
Mr              982.72                    982.76
Dx,g cm-3       1.653                     1.654
Z               1                         1
Mu (mm-1)       4.552                     4.554
F000            484.0                     484.0
F000'           482.05
h,k,lmax        8,8,11                    8,8,11
Nref            1644                      1628
Tmin,Tmax       0.652,0.727               0.610,0.740
Tmin'           0.573

Correction method= # Reported T Limits: Tmin=0.610 Tmax=0.740
AbsCorr = MULTI-SCAN

Data completeness= 0.990          Theta(max)= 19.210

R(reflections)= 0.0563( 1192)     wR2(reflections)= 0.1599( 1628)

S = 1.001                 Npar= 220
```

The following ALERTS were generated. Each ALERT has the format
test-name_ALERT_alert-type_alert-level.
Click on the hyperlinks for more details of the test.

Alert level A

```
THETM01_ALERT_3_A  The value of sine(theta_max)/wavelength is less than 0.550
            Calculated sin(theta_max)/wavelength =    0.4629
```

Author Response: All measured crystals were weakly diffracting and of low quality. Therefore, no reflections at higher theta angle were detected.;

```
PLAT023_ALERT_3_A Resolution (too) Low [sin(theta)/Lambda < 0.6]..      19.21 Degree
PLAT999_ALERT_1_A SHELXL LIST 6 Fo/Fc Unsuitable for FCF-Validation         ! Error
```

Alert level B

```
REFNR01_ALERT_3_B  Ratio of reflections to parameters is < 8 for a
            centrosymmetric structure
            sine(theta)/lambda                0.4629
            Proportion of unique data used    1.0000
            Ratio reflections to parameters   7.4000
PLAT088_ALERT_3_B Poor Data / Parameter Ratio ....................       7.40 Note
PLAT193_ALERT_1_B Cell and Diffraction Temperatures Differ by ....          7 Degree
PLAT342_ALERT_3_B Low Bond Precision on  C-C Bonds ...............    0.02526 Ang.
PLAT922_ALERT_1_B wR2 in the CIF and FCF Differ by ...............     0.0409 Check
```

Alert level C

```
PLAT048_ALERT_1_C MoietyFormula Not Given (or Incomplete) ........     Please Check
PLAT148_ALERT_3_C s.u. on the      a  - Axis is (Too) Large ....      0.020 Ang.
PLAT148_ALERT_3_C s.u. on the      b  - Axis is (Too) Large ....      0.020 Ang.
PLAT148_ALERT_3_C s.u. on the      c  - Axis is (Too) Large ....      0.030 Ang.
PLAT234_ALERT_4_C Large Hirshfeld Difference O1     -- C1          0.22 Ang.
PLAT242_ALERT_2_C Low    'MainMol' Ueq as Compared to Neighbors of      C14 Check
PLAT242_ALERT_2_C Low    'MainMol' Ueq as Compared to Neighbors of      C19 Check
PLAT906_ALERT_3_C Large K Value in the Analysis of Variance ......      2.333 Check
PLAT911_ALERT_3_C Missing FCF Refl Between Thmin & STh/L=  0.463        18 Report
PLAT923_ALERT_1_C S    Values in the CIF and FCF Differ by .......     -0.421 Check
```

Alert level G

```
PLAT005_ALERT_5_G No Embedded Refinement Details Found  in the CIF     Please Do !
PLAT154_ALERT_1_G The s.u.'s on the Cell Angles are Equal ..(Note)       0.02 Degree
PLAT200_ALERT_1_G Reported   _diffrn_ambient_temperature ..... (K)        293 Check
PLAT909_ALERT_3_G Percentage of I>2sig(I) Data at Theta(Max) Still        54% Note
PLAT910_ALERT_3_G Missing # of FCF Reflection(s) Below Theta(Min).          1 Note
PLAT961_ALERT_5_G Dataset Contains no Negative Intensities .......     Please Check
```

3 **ALERT level A** = Most likely a serious problem - resolve or explain
5 **ALERT level B** = A potentially serious problem, consider carefully
10 **ALERT level C** = Check. Ensure it is not caused by an omission or oversight
6 **ALERT level G** = General information/check it is not something unexpected

```
 7 ALERT type 1 CIF construction/syntax error, inconsistent or missing data
 2 ALERT type 2 Indicator that the structure model may be wrong or deficient
12 ALERT type 3 Indicator that the structure quality may be low
 1 ALERT type 4 Improvement, methodology, query or suggestion
 2 ALERT type 5 Informative message, check
```

checkCIF publication errors

Alert level A

```
PUBL003_ALERT_1_A  The contact author’s name is missing,
            _publ_contact_author_name.
PUBL005_ALERT_1_A  _publ_contact_author_email, _publ_contact_author_fax and
            _publ_contact_author_phone are all missing.
            At least one of these should be present.
PUBL006_ALERT_1_A  _publ_requested_journal is missing
            e.g. ’Acta Crystallographica Section C’
PUBL009_ALERT_1_A  _publ_author_name is missing. List of author(s) name(s).
PUBL010_ALERT_1_A  _publ_author_address is missing. Author(s) address(es).
```

```
   5 ALERT level A = Data missing that is essential or data in wrong format
   0 ALERT level G = General alerts. Data that may be required is missing
```

Publication of your CIF

You should attempt to resolve as many as possible of the alerts in all categories. Often the minor alerts point to easily fixed oversights, errors and omissions in your CIF or refinement strategy, so attention to these fine details can be worthwhile. In order to resolve some of the more serious problems it may be necessary to carry out additional measurements or structure refinements. However, the nature of your study may justify the reported deviations from journal submission requirements and the more serious of these should be commented upon in the discussion or experimental section of a paper or in the "special_details" fields of the CIF. *checkCIF* was carefully designed to identify outliers and unusual parameters, but every test has its limitations and alerts that are not important in a particular case may appear. Conversely, the absence of alerts does not guarantee there are no aspects of the results needing attention. It is up to the individual to critically assess their own results and, if necessary, seek expert advice.

If level A alerts remain, which you believe to be justified deviations, and you intend to submit this CIF for publication in a journal, you should additionally insert an explanation in your CIF using the Validation Reply Form (VRF) below. This will allow your explanation to be considered as part of the review process.

Validation response form

Please find below a validation response form (VRF) that can be filled in and pasted into your CIF.

```
# start Validation Reply Form
_vrf_PUBL003_GLOBAL
;
PROBLEM: The contact author’s name is missing,
RESPONSE: ...
;
_vrf_PUBL005_GLOBAL
;
PROBLEM: _publ_contact_author_email, _publ_contact_author_fax and
RESPONSE: ...
;
_vrf_PUBL006_GLOBAL
```

```
;
PROBLEM: _publ_requested_journal is missing
RESPONSE: ...
;
_vrf_PUBL009_GLOBAL
;
PROBLEM: _publ_author_name is missing. List of author(s) name(s).
RESPONSE: ...
;
_vrf_PUBL010_GLOBAL
;
PROBLEM: _publ_author_address is missing. Author(s) address(es).
RESPONSE: ...
;
_vrf_PLAT023_I
;
PROBLEM: Resolution (too) Low [sin(theta)/Lambda < 0.6]..      19.21 Degree
RESPONSE: ...
;
_vrf_PLAT999_I
;
PROBLEM: SHELXL LIST 6 Fo/Fc Unsuitable for FCF-Validation         ! Error
RESPONSE: ...
;
# end Validation Reply Form
```

If you wish to submit your CIF for publication in Acta Crystallographica Section C or E, you should upload your CIF via the web. If you wish to submit your CIF for publication in IUCrData you should upload your CIF via the web. If your CIF is to form part of a submission to another IUCr journal, you will be asked, either during electronic submission or by the Co-editor handling your paper, to upload your CIF via our web site.

PLATON version of 13/08/2017; check.def file version of 08/11/2017

Datablock I - ellipsoid plot

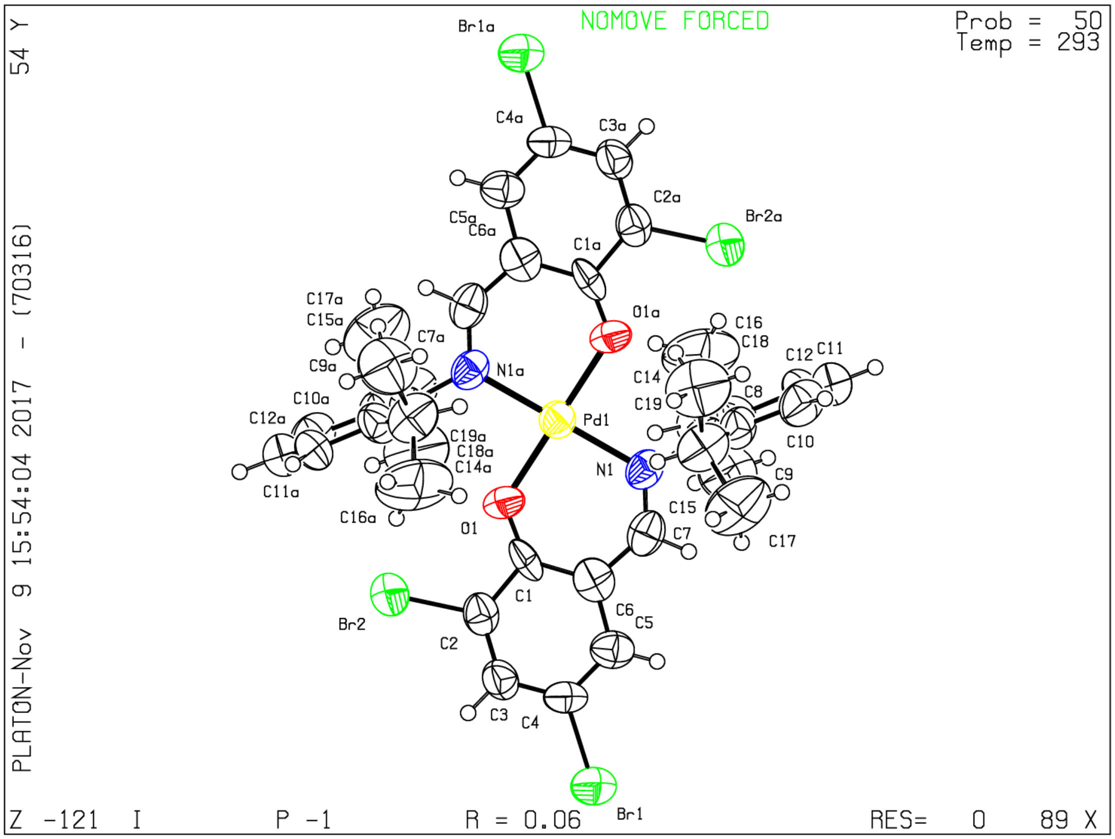

Supplement: Supplementary file 2 — Supplementary material 2 (PDF 150 kb) [file 706_2017_2119_MOESM2_ESM.pdf]
